# Optimal Medical Therapy Targeting Metabolic Status for Secondary Prevention in Patients Undergoing Percutaneous Coronary Intervention

**DOI:** 10.3390/jcm15031108

**Published:** 2026-01-30

**Authors:** Imma Forzano, Viviana Narciso, Mario Enrico Canonico, Domenico Simone Castiello, Domenico Florimonte, Lina Manzi, Federica Semplice, Donato Maria Vallone, Stefano Cristiano, Alessandra Spinelli, Dario D’Alconzo, Roberta Paolillo, Giuseppe Giugliano, Arturo Cesaro, Felice Gragnano, Paolo Calabrò, Giovanni Esposito, Giuseppe Gargiulo

**Affiliations:** 1 Department of Advanced Biomedical Sciences, Federico II University of Naples, 80131 Naples, Italy; imma.forzano@gmail.com (I.F.); viviana.narciso@gmail.com (V.N.); marioenrico.canonico@unina.it (M.E.C.); ds.castiello@gmail.com (D.S.C.); florimontedomenico@gmail.com (D.F.); lina.manzi93@gmail.com (L.M.); federicasemplic@gmail.com (F.S.); donatovallone@outlook.it (D.M.V.); stefano.cristiano@unina.it (S.C.); alessandra.spinelli@unina.it (A.S.); dario.dalconzo@gmail.com (D.D.); robe.paolillo@gmail.com (R.P.); giuseppe.giugliano@unina.it (G.G.); espogiov@unina.it (G.E.); 2 CPC Clinical Research, Department of Medicine, University of Colorado, Aurora, CO 80045, USA; 3 Department of Translational Medical Sciences, University of Campania “Luigi Vanvitelli”, 80138 Naples, Italy; arturo.cesaro@unicampania.it (A.C.); felice.gragnano@unicampania.it (F.G.); paolo.calabro@unicampania.it (P.C.); 4 Division of Cardiology, A.O.R.N. “Sant’Anna e San Sebastiano”, 26100 Caserta, Italy

**Keywords:** coronary artery disease (CAD), secondary prevention, percutaneous coronary intervention (PCI), acute coronary syndrome (ACS), chronic coronary syndrome (CCS), metabolic status, obesity, diabetes

## Abstract

Cardiovascular disease remains the leading cause of death and disability worldwide. Coronary artery disease is the most common clinical manifestation. The underlying pathology is largely attributable to atherosclerosis, a chronic inflammatory process that results in the development of atherosclerotic plaques. Coronary artery disease may present as chronic coronary syndrome or acute coronary syndrome, depending on the extent and stability of plaque disruption. The risk of cardiovascular events is modulated by established clinical and metabolic factors, and risk stratification frameworks are provided by international guidelines. Patients undergoing percutaneous coronary intervention are classified as being at very high cardiovascular risk, despite improvements in invasive and non-invasive management. In this population, the residual ischemic risk remains a significant concern, underscoring the need for targeted secondary prevention strategies. The secondary prevention addresses modifiable risk factors, including hypertension, dyslipidemia, diabetes mellitus, overweight and obesity, and tobacco use, along with recommendations for lifestyle modification. Nonetheless, current interventions leave a substantial proportion of patients exposed to future events, indicating a persistent unmet therapeutic need. Emerging evidence highlights the critical roles of lipid metabolism, inflammation, and metabolic dysfunction in the residual risk pathophysiology. This narrative review aims to examine pharmacological strategies targeting metabolic pathways, notably obesity and diabetes, in the context of secondary cardiovascular prevention in patients undergoing percutaneous coronary intervention.

## 1. Introduction

Cardiovascular disease (CVD) remains the leading cause of mortality and morbidity globally, despite advances in invasive and non-invasive management [1]. Atherosclerosis, the key pathophysiologic mechanism driving ischemic cardiovascular conditions, results from a complex interplay of lipid accumulation and chronic inflammation that promotes plaque formation within the epicardial coronary arteries [2]. Within this framework, coronary artery disease (CAD) constitutes the most prevalent clinical manifestation of atherosclerosis and a major contributor to cardiovascular morbidity and mortality. Clinically, CAD manifests as ischemic heart disease, encompassing both chronic coronary syndrome (CCS) and acute coronary syndrome (ACS). According to the European Society of Cardiology (ESC) guidelines, CCS refers to the stable clinical manifestation of CAD and includes both structural and functional abnormalities involving the epicardial arteries or the coronary microvasculature, resulting in impaired perfusion and myocardial ischemia [3]. In contrast, ACS describes an acute clinical event precipitated by atherosclerotic plaque destabilization, characterized by plaque rupture, fissuring, or thrombus formation, ultimately leading to myocardial infarction (MI) [4].

Although contemporary revascularization strategies, particularly percutaneous coronary intervention (PCI), effectively restore coronary blood flow and alleviate ischemia, they do not address the underlying atherosclerotic disease process. Consequently, secondary prevention targeting residual cardiovascular (CV) risk remains a cornerstone of long-term management in patients with established CAD.

Obesity, defined as a body mass index (BMI) greater than 30 kg/m^2^, encompasses a multifactorial accumulation of adipose tissue and is strongly associated with clinical outcomes [5]. Approximately 70% of obesity-related deaths are attributable to CV causes [6]. Much of the increased CV risk associated with obesity can be attributed to coexisting metabolic disorders, such as insulin resistance and type 2 diabetes mellitus (T2DM) [7]. However, both obesity and overweight are independently associated with increased risk of CAD and cerebrovascular disease, regardless of the presence of metabolic disease [8]. In addition, obesity induces a chronic pro-inflammatory metabolic state that leads to atherosclerotic cardiovascular disease (ASCVD) [9].

Patients with diabetes mellitus (DM) are at particularly high risk for CVD. DM is characterized by hyperglycemia, insulin resistance, and elevated circulating free fatty acids (FFAs), a combination that drives vascular inflammation, endothelial dysfunction, vasoconstriction, thrombosis, and ultimately, atherogenesis leading to CAD [10]. Even after PCI, and regardless of dual antiplatelet therapy (DAPT) regimen, patients with DM remain at a higher risk than those without DM [11]. Therefore, the secondary prevention in such a high-risk population warrants particular attention. Comprehensive management through optimal medical therapy (OMT), encompassing antithrombotic, anti-inflammatory, lipid-lowering, and metabolic therapies, alongside coronary revascularization, is fundamental to optimizing outcomes in patients with CCS or ACS [3,4]. As previously described [12], despite coronary revascularization as well as various regimens of antithrombotic therapies [13,14,15,16,17,18,19,20,21,22,23,24,25,26], these patients remain at high residual risk of CV events during follow-up; thus, there is growing interest in the secondary prevention strategies. However, real-world evidence consistently demonstrates substantial underutilization of guideline-directed medical therapy (GDMT) in patients with CAD [27,28,29].

In a recent review on the same Special Issue, we summarized the most recent evidence about secondary prevention focusing on OMT, targeting lipids and inflammation for patients undergoing PCI [12]. The aim of this narrative review is to summarize and critically appraise the current evidence on pharmacological strategies targeting metabolic pathways, specifically obesity and diabetes, in the context of secondary cardiovascular prevention in patients undergoing PCI. By focusing on post-PCI populations and residual cardiovascular risk, this review seeks to integrate metabolic, inflammatory, and atherosclerotic perspectives, thereby providing a clinically oriented framework that extends beyond weight reduction or glycemic control alone and addresses unmet needs in contemporary post-PCI management.

## 2. Pathophysiology

### 2.1. Pathophysiology of Diabetes Mellitus

**Figure 1:**
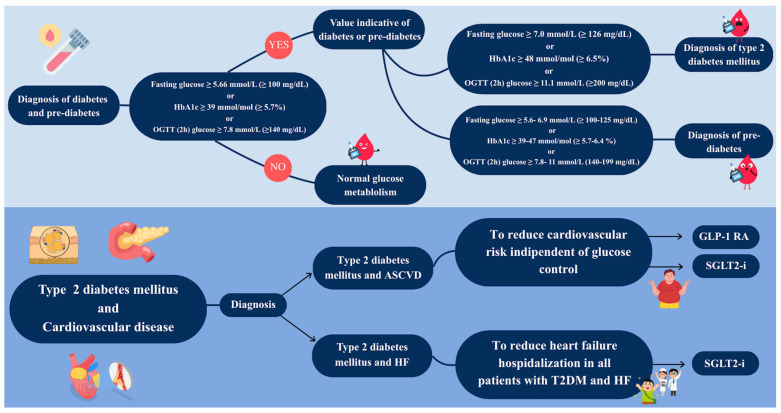
Diagnosis of diabetes and pre-diabetes followed by management of CVD in patients affected by T2DM. ASCVD: atherosclerotic cardiovascular disease, GLP-1 RA: glucagon-like peptide-1 receptor agonist, HbA1c: glycated hemoglobin, HF: heart failure, OGTT: oral glucose tolerance test, SGLT2-i: sodium–glucose co-transporter-2 inhibitors.

DM is a glucose homeostasis disorder. Glucose is both underutilized and overproduced due to inappropriate gluconeogenesis and glycogenolysis, causing hyperglycemia [30]. Glucose homeostasis is strictly regulated by two hormones: glucagon, secreted by pancreatic α-cells, and insulin, secreted by pancreatic β-cells. Elevated blood glucose levels trigger insulin release, facilitating glucose uptake into peripheral tissues, most notably the liver, skeletal muscle, and adipose tissue [31]. There are several types of DM (type 1, type 2, gestational, and others). In non-pregnant patients, the diagnosis of DM is made according to plasma glucose levels and/or glycated hemoglobin (HbA1c) criteria. Notably, DM diagnosis can be possible with a Fasting Plasma Glucose (FPG) ≥ 126 mg/dL (≥7.0 mmol/L) or by performing a 75 g oral glucose tolerance test (OGTT) with a FPG ≥ 200 mg/dL (≥11.1 mmol/L) after 2 h. Alternatively, the diagnosis is made with an HbA1c ≥ 6.5% (≥48 mmol/mol) (Figure 1). DM may also be detected in an individual with a hyperglycemic crisis and plasma glucose ≥ 200 mg/dL, regardless of the last meal [32].

Beyond hyperglycemia per se, insulin resistance represents a key pathophysiological hallmark of type 2 DM, characterized by impaired insulin signaling at the level of skeletal muscle, liver, and adipose tissue, leading to compensatory hyperinsulinemia and progressive β-cell dysfunction. This metabolic milieu promotes lipid abnormalities, endothelial dysfunction, and chronic low-grade inflammation, all of which are central mechanisms in atherogenesis [31].

Hyperglycemia caused by DM leads to chronic systemic inflammation. Moreover, increased intracellular glucose levels activate multiple metabolic pathways that increase reactive oxygen species (ROS) production, resulting in oxidative stress. ROS-mediated activation of protein kinase C-β and increased flux through the aldose reductase pathway contribute to endothelial dysfunction and the development of a pro-inflammatory status [33]. Advanced Glycation End-Products (AGE) are compounds generated in hyperglycemia and not well metabolized. AGE accumulates in patients with suboptimal levels of glycemia for a long time, leading to microvascular damage. AGE interacts with its specific receptors (RAGE) expressed on endothelial cells, macrophages, and vascular smooth muscle cells, triggering nuclear factor-κB (NF-κB) signaling, cytokine release, and vascular inflammation, thereby accelerating both microvascular and macrovascular disease [34]. Microvascular diseases in DM are represented by nephropathy, retinopathy, and neuropathy [35,36]. Soluble IL-2 receptor (sIL2r), a marker of T cell activation, is elevated in numerous inflammatory diseases. It has been demonstrated that sIL2r concentration is elevated in DM. This is related to a premature calcification grade. Therefore, DM generates oxidative stress, causing endothelial dysfunction, arterial stiffness, and pro-thrombotic status [37,38]. At the macrovascular level, diabetes is associated with a diffuse and accelerated form of atherosclerosis, characterized by endothelial nitric oxide depletion, enhanced platelet reactivity, impaired fibrinolysis, and a pro-thrombotic state, which are particularly relevant in patients undergoing PCI [34]. Thus, patients with DM develop premature atherosclerosis with microvascular damage, increasing the risk of ASCVD [39,40,41,42]. Real-world data show that DM is strictly related to CVD [43]. In particular, CVD causes the 55% of deaths in patients with DM [44,45]. Nonetheless, routine screening for CVD in asymptomatic individuals with DM is not recommended [46]. Hyperglycemia demonstrated to be related to worse clinical outcomes in patients with ACS [38,47]. Intriguingly, DM is related to a higher rate of In-Stent Restenosis (ISR). Mechanistically, hyperglycemia and insulin resistance promote neointimal hyperplasia through smooth muscle cell proliferation, extracellular matrix deposition, and impaired endothelial repair, providing a biological explanation for the increased risk of ISR in diabetic patients [48]. It has been demonstrated that adequate control of risk factors in patients with DM, including optimal management of glycemic levels, can reduce the risk of death, MI, or stroke comparable with the general population [49]. On the contrary, poor glycemic control, defined by HbA1c ≥ 6.5% (≥48 mmol/mol), was associated with a higher risk of stent failure driven by ISR in a wide cohort of DM patients undergoing PCI in Sweden [50]. This underlines the importance of strict glycemic control after PCI.

### 2.2. Pathophysiology of Obesity

Obesity (and overweight) is defined as an excessive accumulation of fat within adipose tissue (AT) [51]. It is a complex, multifactorial condition arising from a chronic imbalance between energy intake and energy expenditure. It is influenced by a combination of neurobiological mechanisms, genetic predisposition, socioeconomic status, and psychological factors. From a pathophysiological standpoint, obesity is increasingly recognized as a systemic metabolic disease rather than a simple disorder of excess weight, with profound effects on endocrine, inflammatory, and cardiovascular homeostasis. Obesity is actually considered an epidemic. It is estimated that almost 23% of adults in European countries are obese [6]. Moreover, it has been observed that the prevalence has more than doubled over the past 40 years [52]. According to the World Health Organization (WHO), obesity can be classified using Body Mass Index (BMI), which is based on the ratio of a person’s weight and height expressed in kilograms (kg) and meters cube (m^2^), respectively. WHO classification of obesity in adults considers normal weight a BMI from 18.5 to 24.9 kg/m^2^, overweight a BMI from 25 to <30 kg/m^2^, and obesity a BMI ≥ 30 kg/m^2^. Obesity can be further classified according to its severity: obesity class 1 with a BMI from 30 to 34 kg/m^2^, obesity class 2 with a BMI from 35 to 39 kg/m^2^, and obesity class 3 (severe obesity) with a BMI ≥ 40 kg/m^2^. There are some country-specific cut-offs according to population, for example, for Asian people, or age, or particular conditions (pregnancy) [6]. While an increased risk of mortality is well established among underweight individuals (BMI < 18.5 kg/m^2^), evidence in those with BMI > 25 kg/m^2^ indicates a progressively increasing risk of all-cause mortality with higher BMI values. Notably, nearly 70% of obesity-related deaths are attributable to CVD [53,54]. Furthermore, high BMI is related to common CV risk factors such as T2DM, dyslipidemia, and hypertension, but it is also a strong independent risk factor for CAD, HF, atrial fibrillation, and stroke [55,56,57]. In a pathophysiological view, excess adiposity contributes to CVD through multiple mechanisms, including insulin resistance, sympathetic nervous system activation, renin–angiotensin–aldosterone system upregulation, and chronic low-grade inflammation, all of which promote endothelial dysfunction and atherosclerotic plaque development [56]. Notably, severe obesity with BMI ≥ 40 kg/m^2^ has been associated with higher in-hospital mortality post-PCI, particularly in ST-elevation myocardial infarction (STEMI) patients and with higher rates of contrast-induced nephropathy, nephropathy requiring dialysis, and vascular complications compared with overweight patients [58,59].

Interestingly, an “obesity paradox” has been documented where a higher BMI is associated with improved survival rate in patients with heart failure (HF) compared to those with a normal BMI. Several pathophysiological mechanisms have been proposed to explain this phenomenon, including greater metabolic and nutritional reserves, reduced circulating levels of natriuretic peptides, altered neurohormonal activation, and a potentially more favorable inflammatory profile in overweight patients compared with lean individuals affected by chronic disease. In addition, BMI may inadequately reflect body composition, failing to distinguish between lean mass and fat mass, thereby misclassifying patients with higher muscle mass and more preserved functional status [60]. However, trials including glucagon-like peptide-1 receptor agonists (GLP-1RA) aimed to reduce body weight have demonstrated that the reduction in body weight improves CV outcomes. This apparent discrepancy suggests that the obesity paradox may reflect residual confounding, reverse causation, or limitations of BMI as a surrogate of adiposity, rather than a true protective effect of excess fat mass. This evidence makes it clear that there is a need to improve our knowledge of the mechanisms linking adiposity, fat distribution, and metabolic dysfunction to cardiovascular outcomes. A more precise characterization of adiposity phenotypes may provide further evidence [61].

**Figure 2:**
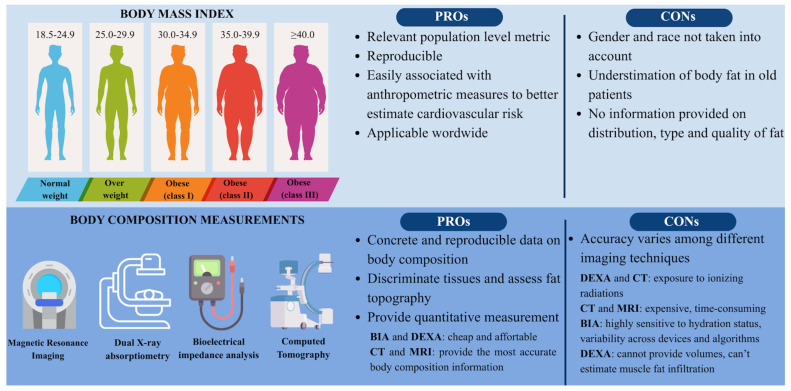
BMI is a simple and reproducible anthropometric measure that is widely accessible. However, BMI does not account for several important variables, including ethnicity, sex, and most critically, body composition, such as fat mass and lean mass distribution. Alternative modalities for assessing body composition include BIA, CT, DEXA, and MRI. These techniques offer objective and reproducible data on body composition. Nonetheless, each method has limitations. The accuracy of measurements may vary depending on the technique used; CT and DEXA involve exposure to ionizing radiation; DEXA does not provide information on tissue volumes or intramuscular fat infiltration and may yield less accurate results in individuals with severe obesity. Additionally, both CT and MRI are costly and not universally available, while BIA is influenced by the individual’s hydration status. BIA: bioelectrical impedance analysis, BMI: body mass index, CT: computed tomography, DEXA: dual-energy X-ray absorptiometry, MRI: magnetic resonance imaging.

BMI is a simple, reproducible, and widely used parameter. However, BMI does not take into consideration relevant factors (such as ethnicity, sex, and age), but primarily does not take into account body composition, such as the percentage of body fat. BIA (bioelectrical impedance analysis), CT (computed tomography), DEXA (dual-energy X-ray absorptiometry), and MRI (magnetic resonance imaging) can provide further information on body composition. However, there are some limitations: accuracy varies among different techniques, CT and DEXA expose patients to ionizing radiations, DEXA does not provide volumes or muscle fat infiltration, and results are less accurate in severely obese patients, CT and MRI are expensive and not available worldwide, and finally, BIA is sensitive to hydration status (Figure 2) [62].

According to the ESC clinical consensus statement on coronary pathophysiology and microcirculation, different types of adipose tissue (AT) can be identified. AT is generally classified as subcutaneous adipose tissue (SAT), located in the hypodermis across thoracic, abdominal, gluteal, and femoral areas, as well as visceral adipose tissue (VAT), located within the abdomen and thorax. VAT can be further sub-classified into thoracic adipose tissue (ThAT), including pericardial, non-pericardial, epicardial, and perivascular fat, and abdominal AT, comprising intra- and retroperitoneal depots. Perivascular adipose tissue (PVAT) includes the AT localized around a vessel at a radial distance equal to that between two adjacent vessels. If the vessel has a diameter > 2 cm, the PVAT is the AT localized at 2 cm from the outer vessel wall. AT is known to secrete adipokines, bioactive molecules that can influence CV physiology, working on endocrine pathways [63].

Thus, the ESC clinical consensus statement classification is useful to highlight biological differences within AT. Indeed, VAT is formed by smaller and less differentiated adipocytes with a higher concentration of macrophages compared to SAT. This determines that VAT is metabolically active in secreting proinflammatory adipokines, leading to insulin resistance. Thus, VAT is associated with atherosclerotic process and ASCVD due to obesity-induced low-grade inflammation [9]. VAT-derived adipokines, such as leptin, resistin, and pro-inflammatory cytokines, directly contribute to vascular inflammation, oxidative stress, and endothelial dysfunction, reinforcing the link between central obesity and CAD [63]. On the other hand, PVAT is rich in various cell types, including adipocytes, which results in small and few lipid droplet accumulation, neuronal cells, immune cells, and stromal vascular fraction (preadipocytes, fibroblasts, endothelial cells, and mesenchymal stem cells) that could be involved in the atherogenic process [64]. Furthermore, it has been discovered that PVAT secretes adipokines, anti- and pro-inflammatory factors with a direct paracrine effect on arterial walls and contributes to vascular homeostasis [65]. Patients affected by obesity have elevated levels of proinflammatory cytokines and hsCRP. Intriguingly, obesity is related to higher platelet activity demonstrated in ex vivo studies, probably due to the chronic proinflammatory status that obesity is connected with. This pro-thrombotic and pro-inflammatory environment may adversely affect procedural and long-term outcomes after PCI by promoting platelet hyperreactivity, impaired endothelial healing, and restenosis [66,67].

Therefore, obesity is strictly related to the development of ASCVD, both with obstructive CAD and microcirculation disease [9]. The relationship between obesity and ASCVD is partly mediated by the strong association between obesity and the development of CV risk factors, such as DM, hypertension, and dyslipidemia, and partly by obesity and overweight themselves, which are recognized as independent risk factors [8,68,69]. It is clear that the importance of AT and its relevance to control obesity and CV risk factors, especially in secondary prevention of ASCVD.

## 3. Secondary Prevention

### 3.1. Diabetes Mellitus

#### 3.1.1. Glucagon-like Peptide 1 Receptor Agonists for Diabetes Mellitus

**Figure 3:**
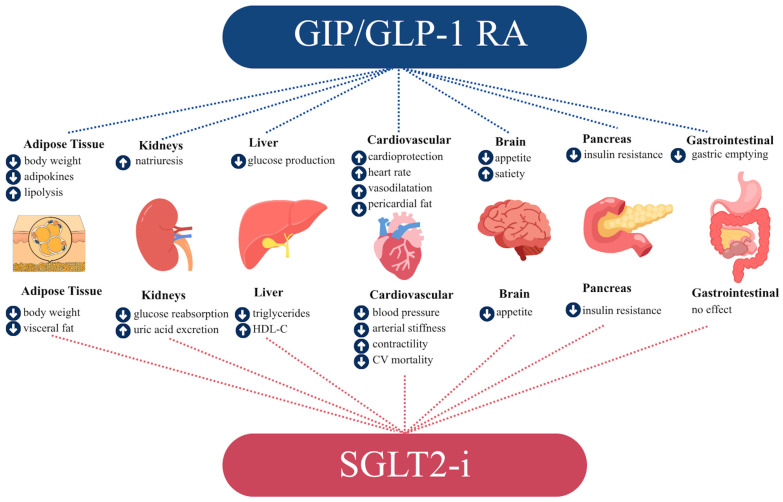
GLP-1RA and GIP exert their effects by enhancing the action of incretin hormones, which are secreted by the intestinal epithelium in response to nutrient ingestion. These agents promote glucose-dependent insulin secretion from pancreatic β-cells and reduce glucose production in the liver, reducing insulin resistance, thereby contributing to the maintenance of glucose homeostasis. In addition to their insulinotropic effects, GLP-1RA and GIP also delay gastric emptying, reduce gastrointestinal motility, suppress appetite, increase natriuresis. They ameliorate cardioprotection, heart rate, and vasodilation; moreover, they reduce pericardial fat. SGLT2-i exert their primary therapeutic effect in diabetes management by promoting glucosuria. Beyond glycemic control, these agents confer CV benefits through favorable modulation of several key parameters, including reductions in blood pressure, systemic inflammation, oxidative stress, and renal injury. These effects are related to the intrinsic glucosuric and natriuretic properties of this class of medications, as well as metabolic shifts that favor ketone body utilization over glucose and FFA, an adaptation that may confer an energetic advantage to cardiomyocytes. CV: cardiovascular, GLP-1RA: glucagon-like peptide-1 receptor agonists, HDL-C: High-Density Lipoprotein Cholesterol, SGLT2-i: Sodium–glucose cotransporter 2 inhibitors.

GLP-1RAs act by enhancing the physiological effects of incretins, which are hormones released by the intestine in response to nutrient intake. GLP-1RAs stimulate pancreatic β cells to release insulin, thereby participating in the regulation of glucose homeostasis (Figure 3) [70]. Moreover, incretins delay gastric emptying, decrease intestinal motility, and reduce appetite [71]. Beyond their glucose-lowering effects, GLP-1RAs exert multiple direct and indirect actions on the cardiovascular system that are largely independent of glycemic control. Experimental and clinical data have demonstrated that GLP-1 receptors are expressed in cardiovascular tissues, including endothelial cells, smooth vascular muscle cells, and cardiomyocytes, providing a mechanistic basis for their pleiotropic cardiovascular effects. In this respect, GLP-1RAs show several positive effects on the CV system, including improvement of endothelial function, plaque stabilization, and blood pressure. These effects are mediated through attenuation of vascular inflammation, reduction in oxidative stress, improvement of endothelial nitric oxide bioavailability, and modulation of atherosclerotic plaque composition, ultimately translating into improved vascular homeostasis [72,73]. This effect contributes to reducing the residual CV risk in secondary prevention [12].

**Figure 4:**
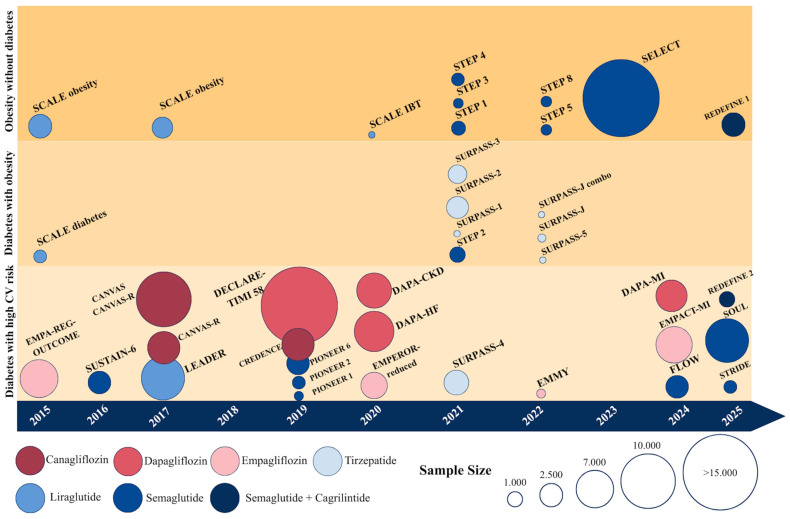
Timeline of landmark studies of the last decade about therapies targeting metabolic status (GLP-1RA, GLP-1RA/GIP, and SGLT2-i), organized according to population investigated (obesity with no diabetes; diabetes with obesity; diabetes with high CV risk). The size of the circles is proportionate to the numerosity of the population studied. CV: cardiovascular, GLP-1RA: glucagon-like peptide 1 receptor agonists, GIP: glucose-dependent insulinotropic polypeptide, SGLT2-i: sodium glucose cotransporter-2 inhibitors.

Numerous trials evaluating CV outcomes in patients with T2DM treated with GLP-1RAs have been conducted. Consistently, large cardiovascular outcome trials have demonstrated that GLP-1RAs significantly reduce major adverse cardiovascular events (MACE) in patients with type 2 diabetes, establishing their role not only as antidiabetic agents but also as disease-modifying therapies in cardiovascular prevention (Figure 4, Table 1).

The LEADER (a long-term, multicenter, international, randomized double-blind, placebo-controlled trial to determine liraglutide effects on CV events) trial analyzed the CV effect of liraglutide on top of DM standard of care. A total of 9340 T2DM patients were randomized to liraglutide or placebo once daily. Liraglutide was associated with a lower rate of death from CV causes, nonfatal MI, or nonfatal stroke than in patients treated with placebo [13.0% of patients in the liraglutide group, 14.9% of patients in the placebo group; hazard ratio (HR) 0.87, 95% confidence interval (CI), 0.78 to 0.97; *p* < 0.001] [74].

The ELIXA (a randomized, double-blind, placebo-controlled, parallel-group, multicenter study to evaluate CV outcomes during treatment with lixisenatide in T2DM patients after an ACS) trial showed that in 6068 patients with T2DM experiencing a recent ACS. Compared to placebo, the addition of lixisenatide to standard of care did not significantly modify the rate of the primary endpoint, including CV death, MI, stroke, or hospitalization for unstable angina (HR 1.02; 95% CI 0.89–1.17). The rate of hospitalization for HF was similar between groups, as well as the rate of CV death [75,76].

The REWIND (the effect of dulaglutide on major cardiovascular events in patients with Type 2 Diabetes: researching cardiovascular events with a weekly INcretin in Diabetes) trial was a double-blind, randomized, placebo-controlled trial that recruited 9901 participants ≥ 50 years old, randomly assigned to dulaglutide 1.5 mg subcutaneously or placebo. Dulaglutide demonstrated a reduction of about 12% the risk of MACE compared to placebo (HR 0.88, 95% CI 0.79 to 0.99; *p* = 0.026) [77].

Moreover, 9463 T2DM patients were randomized to albiglutide administered subcutaneously (30–50 mg) or placebo in the HARMONY (a long-term, randomized, double-blind, placebo-controlled study to determine the effect of albiglutide, when added to standard blood glucose-lowering therapies, on major CV events in patients with T2DM) trial. Albiglutide reduced of 22% the risk of CV events compared to placebo (*p* < 0.0001 for non-inferiority; *p* = 0.0006 for superiority) [78].

In the EXSCEL (exenatide study of cardiovascular event lowering trial, a randomized, placebo-controlled clinical trial to evaluate CV outcomes after treatment with exenatide once weekly in patients with T2DM), study, 14,752 patients with T2DM, with or without previous CV disease, were randomized to receive subcutaneous injections of 2 mg of exenatide or a matching placebo once weekly. MACE rates did not differ significantly between the exenatide group or the placebo group (exenatide group: 11.4%; 3.7 events per 100 person-years; placebo group: 12.2%; 4.0 events per 100 person-years; HR 0.91, 95% CI, 0.83 to 1.00; *p* = 0.06 for superiority) [79].

The AMPLITUDE-O (a randomized, double-blind, placebo-controlled, parallel-group, multicenter study to evaluate the effect of efpeglenatide on CV Outcomes in T2DM patients at high CV risk) trial randomized 4076 participants with T2DM and a history of CV disease or kidney disease to receive 4 or 6 mg efpeglenatide subcutaneously weekly or placebo. Compared to placebo, efpeglenatide demonstrated a reduction in CV events by 27% (HR 0.73, 95% CI, 0.58 to 0.92; *p* < 0.001 for noninferiority) [80].

More recently, semaglutide emerged as an effective and safe GLP-1RAs administrable once weekly [81]. In the SUSTAIN-6 (a long-term, randomized, double-blind, placebo-controlled, multinational, multicenter trial to evaluate cardiovascular and other long-term outcomes with semaglutide in subjects with Type 2 Diabetes) trial, 3297 patients at high CV risk affected by T2DM were randomly assigned to receive semaglutide or placebo subcutaneously once weekly on top of therapy for diabetes for 104 weeks. Semaglutide resulted non-inferior to placebo for the composite outcome of CV death, nonfatal MI, or nonfatal stroke, with lower rates in the semaglutide group compared to the placebo group (6.6% vs. 8.9%; HR 0.74, 95% CI, 0.58 to 0.95; *p* < 0.001 for noninferiority) [82].

Notably, semaglutide is the first GLP-1RA available for oral administration [83]. Oral administration of semaglutide demonstrated efficacy and safety. In the PIONEER-6 (a trial investigating the CV safety of oral semaglutide in subjects with T2DM) trial, oral semaglutide administered once daily reduced CV outcomes compared to placebo (MACE: 3.8% in the oral semaglutide group and 4.8% in the placebo group; HR 0.79; 95% CI, 0.57 to 1.11; *p* < 0.001 for noninferiority). However, treatment discontinuation due to gastrointestinal adverse events was more frequent in the semaglutide versus the placebo group [84]. Intriguingly, recent evidence suggests that semaglutide may reduce the risk of kidney events and CV death in patients with T2DM and chronic kidney disease (CKD) [85,86].

The efficacy of oral semaglutide in CV outcomes was demonstrated in the SOUL (Semaglutide CV Outcomes Trial in Patients With T2DM) trial. In this double-blind, placebo-controlled, event-driven, superiority trial, 9650 patients with T2DM and ASCVD, CKD, or both were randomized to oral semaglutide or placebo administered daily. Semaglutide was associated with a significantly lower risk of MACE than placebo (12.0% vs. 13.8%, HR, 0.86, 95% CI, 0.77 to 0.96; *p* = 0.006). An increase in the incidence of serious adverse events was not observed when comparing semaglutide and placebo [87].

The STRIDE trial was the first phase 3 study to evaluate a GLP-1RA in patients with peripheral artery disease (PAD) and T2DM presenting with claudication (Fontaine stage IIa). After 52 weeks of treatment, semaglutide significantly increased the maximum walking distance compared with placebo, with an estimated treatment ratio of 1.13 (95% CI 1.06–1.21), corresponding to a mean improvement of 39.9 m versus placebo. In addition, semaglutide significantly improved pain-free walking distance, quality of life (assessed by two validated instruments), ankle-brachial index, and, as an exploratory endpoint, reduced the composite of rescue therapy or all-cause death compared with placebo [88].

Interestingly, semaglutide showed the ability to reduce inflammation in patients affected by T2DM, reaching a reduction of 37% of hsCRP levels compared to placebo and even compared to empagliflozin [83,89,90].

GLP-1RAs were associated with better left ventricular ejection fraction (LVEF) and reduced infarct size (IS) in patients with MI undergoing PCI, lowering the CV risk of morbidity and mortality [91].

Therefore, in secondary prevention, the most recent ESC guidelines on CCS recommend GLP-1RAs with proven CV benefit (dulaglutide, efpeglenatide, liraglutide, and semaglutide) in patients with T2DM and established ASCVD. This recommendation includes a Class I, Level A, and applies irrespective of baseline or target HbA1c levels and independently of concomitant glucose-lowering therapies, with the primary goal of reducing CV events [3,92].

#### 3.1.2. Glucagon-like Peptide 1 Receptor Agonists and Glucose-Dependent Insulinotropic Polypeptide for Diabetes Mellitus

Tirzepatide is an emerging and promising agent currently in the spotlight. It is considered the first “twincretin,” as it acts as both a glucose-dependent insulinotropic polypeptide (GIP) receptor agonist and a GLP-1RA, thereby harnessing the complementary effects of both incretin pathways [93].

The SURPASS (study of tirzepatide in participants with T2DM not controlled with diet and exercise alone) program investigated tirzepatide in patients with T2DM. Although these trials did not specifically enroll post-PCI patients, the observed metabolic and CV benefits are highly relevant in terms of secondary prevention. In particular, SURPASS-1 (a randomized, double-blind, placebo-controlled trial comparing the efficacy and safety of three tirzepatide doses versus placebo in patients with T2DM, inadequately controlled with diet and exercise alone) demonstrated the superiority of tirzepatide over placebo in patients with T2DM inadequately controlled by lifestyle [94] achieving HbA1c reductions leading to hypoglycemia for 31–52% of participants. Weight loss ranged between 7 and 9.5 kg, without an increased incidence of severe adverse events [95,96]. These findings underscore the potential of tirzepatide to optimize glycemic and weight control in high-risk population including those post-PCI patients.

In the SURPASS-2 (a phase 3, randomized, open-label trial comparing efficacy and safety of tirzepatide versus semaglutide once weekly as add-on therapy to metformin in patients with T2DM) trial tirzepatide (in three different dosages of 5, 10, and 15 mg) was compared to semaglutide in T2DM [97]. Tirzepatide achieved a greater HbA1c reduction (−0.15%, *p* = 0.02 with 5 mg, −0.39%, *p* < 0.001 with 10 mg, and −0.45%, *p* < 0.001 with 15 mg vs. semaglutide) and additional weight loss up to 5.5 kg (*p* < 0.001 for all comparisons) [98].

In SURPASS-3 (a randomized, phase 3, open-label trial comparing the effect of LY3298176 versus titrated insulin degludec on glycemic control in patients with T2DM) study tirzepatide (in the three different dosages of 5, 10, and 15 mg) led to a larger HbA1c reduction (−1.93% to −2.37% vs. −1.34%, *p* < 0.0001 for all comparisons) and greater weight loss (−9.8 to −15.2 kg, *p* < 0.0001 for all comparisons) compared with insulin degludec [99]. MRI sub-studies confirmed reductions in liver fat and visceral adiposity [100,101]. Such effects directly target key drivers of recurrent ischemia after PCI.

The SURPASS-4 (efficacy and safety of LY3298176 once weekly versus insulin glargine in patients with T2DM and increased CV risk) trial enrolled high-CV-risk patients with T2DM [102]. Tirzepatide (in the three different dosages of 5, 10, and 15 mg) was associated with a greater improvement of HbA1c (−2.24% to −2.58% vs. −1.44% with insulin glargine, *p* < 0.0001 for all comparisons) and weight (−7.1 to −11.7 kg vs. +1.9 kg, *p* < 0.0001 for all comparisons) compared with insulin glargine. Post hoc analysis showed positive effects of tirzepatide on eGFR decline and albuminuria [103]. This is particularly relevant to secondary prevention post-PCI, where cardiorenal disease accelerates adverse outcomes.

Finally, in SURPASS-5 (randomized, Phase 3, double-blind trial comparing the effect of the addition of tirzepatide versus placebo in patients with T2DM inadequately controlled on insulin glargine with or without metformin) trial the addition of tirzepatide at 5, 10 or 15 mg to insulin glargine improved HbA1c (−2.11% with 5 mg, −2.40% with 10 mg and −2.34% with 15 mg, vs. −0.86% with placebo, *p* < 0.001 for all the dosages) and led to a further weight loss (−5.4 with 5 mg, −7.5 with 10 mg and −8.8 kg with 15 mg compared with +1.6 kg, *p* < 0.001 for all the dosages) [104,105].

Tirzepatide was tested in Japanese patients in the SURPASS J-mono (a phase 3 study of tirzepatide monotherapy compared to dulaglutide 0.75 mg in patients with T2DM) and J-combo (safety and efficacy of tirzepatide as an add-on to single oral antihyperglycemic medication in patients with type 2 diabetes in Japan). In these cohorts, compared to dulaglutide, tirzepatide improved glycemic control on top of oral agents [106,107,108], confirming consistency across populations, supporting global applicability in secondary prevention.

Across the SURPASS (study of tirzepatide in participants with T2DM not controlled with diet and exercise alone) program, tirzepatide consistently achieved robust HbA1c reductions, substantial weight loss, and improvements in visceral adiposity and renal parameters. For patients with T2DM undergoing PCI, these benefits address key determinants of recurrent ischemic events. While dedicated outcome trials in post-PCI populations are lacking, tirzepatide emerges as a promising therapy for secondary prevention beyond conventional glucose-lowering strategies.

The SURPASS-CVOT was a large, event-driven cardiovascular outcomes trial designed to assess the CV safety and efficacy of tirzepatide compared with dulaglutide in patients with type 2 DM and ASCVD [109]. More than 13,000 participants across approximately 30 countries were randomized to receive once-weekly tirzepatide or dulaglutide on top of standard care. The primary endpoint was the composite of MACE, including CV death, nonfatal MI, and nonfatal stroke. Tirzepatide demonstrated noninferiority to dulaglutide for MACE (HR, 0.92; 95% CI, 0.83 to 1.01; *p* = 0.086). Exploratory analyses revealed additional metabolic and renal benefits: participants on tirzepatide experienced markedly greater reductions in HbA1c (−2.1% vs. −1.3%) and body weight (−10.4 kg vs. −3.8 kg) compared with dulaglutide, alongside improvements in albuminuria and estimated glomerular filtration rate decline. The safety profile was consistent with previous incretin studies, with gastrointestinal adverse events (nausea, vomiting, diarrhea) being the most common and leading to modestly higher discontinuation rates in the tirzepatide arm (13.3% vs. 10.2%). No excess in pancreatitis, retinopathy, or cardiovascular adverse events was reported [110].

#### 3.1.3. Sodium Glucose Cotransporter-2 Inhibitors

Sodium Glucose Cotransporter-2 Inhibitors (SGLT2-i), also known as gliflozins, are a pleiotropic group of drugs modulating glucose metabolism [111]. SGLT2 proteins are expressed in the nephron, the functional unit of the kidney, and are responsible for reabsorbing approximately 90% of filtered glucose in the early segment of the proximal convoluted tubule [112]. SGLT2-i are molecules that inhibit SGLT2 activity to reduce glucose reabsorption, leading to increased glycosuria and reduced blood glucose levels. This mechanism of action explains their antidiabetic function [113,114]. SGLT2-i has demonstrated additional CV benefits through mechanisms that are not yet fully understood. The main proposed pathways include improved cardiomyocyte calcium handling, enhanced myocardial energy efficiency, reduced inflammation, induction of autophagy and lysosomal degradation, decreased epicardial fat, and lowering of blood pressure [115]. Furthermore, accumulating evidence indicates that SGLT2-i exert relevant anti-atherosclerotic effects through multiple interconnected pathways. Experimental and translational studies suggest that SGLT2-i attenuate endothelial dysfunction by reducing oxidative stress, improving nitric oxide bioavailability, and suppressing endothelial inflammatory signaling, which are key early drivers of atherosclerosis. Their favorable effects on visceral and epicardial adipose tissue inflammation may also indirectly limit vascular inflammation and plaque progression. Collectively, these pleiotropic actions provide a mechanistic rationale for the observed cardiovascular protection associated with SGLT2-i and support their potential role in mitigating atherosclerotic disease burden and residual cardiovascular risk, including in patients with established coronary artery disease [116].

Several trials have been conducted to evaluate SGLT2-i role in CV disease (Table 2). In EMPA-REG OUTCOME [a Phase III, multicenter, international, randomized, parallel group, double-blind CV safety study of BI 10773 (10 mg and 25 mg administered orally once daily) compared to usual care in Type 2 diabetes mellitus patients with increased cardiovascular risk] A total of 7020 patients with T2DM were randomly assigned to receive 10 mg or 25 mg of empagliflozin or placebo once daily. The primary outcome was a composite of death from CV causes, nonfatal MI, or nonfatal stroke. Compared to placebo, there were significantly lower rates of death from CV causes, hospitalization for HF, and death from any cause in the empagliflozin group (HR, 0.86; 95% CI, 0.74 to 0.99; *p* < 0.001 for noninferiority and *p* = 0.04 for superiority). An increased rate of genital infection was observed in the empagliflozin group. No other adverse events were observed [117].

In the DECLARE-TIMI 58 (dapagliflozin effect on cardiovascular events: a multicenter, randomized, double-blind, placebo-controlled trial to evaluate the effect of dapagliflozin 10 mg once daily on the incidence of cardiovascular death, myocardial infarction, or ischemic stroke in patients with Type 2 Diabetes) trial, the CV safety profile of dapagliflozin was investigated. 17,160 patients with T2DM were randomly assigned to receive dapagliflozin or placebo. Dapagliflozin demonstrated significantly reduction in MACE (95% CI, <1.3; *p* < 0.001 for noninferiority). Moreover, patients in the dapagliflozin group experienced significantly lower rates of the composite endpoint of cardiovascular death or hospitalization for heart failure compared with the placebo group. Treatment with dapagliflozin was also associated with a reduced risk of CV death and hospitalization for HF [118,119,120].

Other SGLT2-i, such as canagliflozin, have also been extensively studied. The CANVAS (a randomized, multicenter, double-blind, parallel, placebo-controlled study of the effects of JNJ-28431754 on CV outcomes in adult subjects with T2DM) and CANVAS-R (a randomized, multicenter, double-blind, parallel, placebo-controlled study of the effects of canagliflozin on renal endpoints in adult subjects with T2DM) trials were combined into the CANVAS program, which analyzed data from 10,142 participants with T2DM and high CV risk randomized to either canagliflozin or placebo. Treatment with canagliflozin resulted in a lower rate of CV death, nonfatal MI, or nonfatal stroke compared with placebo (HR 0.86; 95% CI, 0.75 to 0.97; *p* < 0.001 for noninferiority, *p* = 0.02 for superiority). Adverse events such as genital and urinary tract infections, volume depletion, and increased diuresis were more common in the canagliflozin group. Nevertheless, canagliflozin demonstrated potential renal benefits [121,122,123,124,125,126]. These findings were further confirmed in the CREDENCE (a randomized, double-blind, event-driven, placebo-controlled, multicenter study of the effects of canagliflozin on renal and CV outcomes in subjects with T2DM and diabetic nephropathy) trial, in which 4401 patients with T2DM and diabetic kidney disease were randomized to canagliflozin or placebo. In the canagliflozin group, the risks of kidney failure and major CV events were significantly lower compared with placebo (CI 95%, HR 0.70; *p* < 0.001 global) [127,128].

Ertugliflozin was evaluated in the VERTIS CV (randomized, double-blind, placebo-controlled, parallel-group study to assess CV outcomes following treatment with ertugliflozin in subjects with T2DM and established vascular disease) trial, including 8246 patients with T2DM and ASCVD to receive either ertugliflozin or placebo. The trial demonstrated no differences in MACE comparing ertugliflozin and placebo (11.9% vs. 11.9% in the placebo group; HR, 0.97; 95.6% CI, 0.85 to 1.11) [129,130,131].

Sotagliflozin, a dual SGLT1/2 inhibitor, has been evaluated in patients with previous ASCVD in a secondary analysis of the SCORED (effect of sotagliflozin on cardiovascular and renal events in patients with Type 2 Diabetes and moderate renal impairment who are at cardiovascular risk) trial, which included 10,584 patients with T2DM, CKD, and additional cardiovascular factors. Compared to placebo, sotagliflozin reduced MACE (HR, 0.77; 95% CI, 0.65 to 0.91, *p* = 0.0020), with a significant reduction in MI and stroke. Notably, the benefit was consistent among patients with and without previous CV events [132].

According to the most recent ESC guidelines on ACS, three trials in particular have demonstrated CV benefit in patients with established ASCVD and T2DM: EMPA-REG Outcome, CANVAS PROGRAM, DECLARE TIMI 58, on empagliflozin, canagliflozin, and dapagliflozin, respectively [4,117,120,126]. A meta-analysis demonstrated that SGLT2-i have moderate effect on MACE in patients with established ASCVD. Anyway, SGLT2i shows significant benefit on hospitalization for HF and progression of CKD independently of a history of HF or renal disease [133].

Interestingly, in the EMMY (impact of EMpagliflozin on cardiac function and biomarkers of heart failure in patients with acute MYocardial infarction) trial, which evaluated empagliflozin in patients with a recent MI who underwent PCI within the previous 72 h, empagliflozin significantly reduced NT-proBNP levels over 26 weeks and improved both functional and structural echocardiographic parameters. Specifically, NT-proBNP reduction was 15% greater in the empagliflozin group compared with placebo (95% CI −4.4% to −23.6%, *p* = 0.026), and the LVEF improvement was significantly greater (1.5%; 95% CI 0.2–2.9%; *p* = 0.029) [134].

Recently, two trials on SGLT2-i after ACS have been conducted. In EMPACT-MI (a streamlined, multicentre, randomized, parallel group, double-blind placebo-controlled superiority trial to evaluate the effect of EMPAgliflozin on hospitalization for HF and mortality in patients with aCuTe MI) trial patients hospitalized for MI at risk for HF, on the basis of newly developed left ventricular ejection fraction of <45% or signs or symptoms of congestion, with or without T2DM were randomized to receive empagliflozin at a dose of 10 mg daily or placebo in addition to standard care within 14 days after admission for the acute event. The primary endpoint was a composite of first hospitalization for HF or all-cause death, with a subsequent evaluation of individual components of the primary endpoint. Compared to placebo, the trial demonstrated that empagliflozin did not significantly reduce the primary endpoint [135]. However, it demonstrated that empagliflozin reduced the risk of HF hospitalizations (3.6% vs. 4.7% with events; HR: 0.77, 95% CI 0.60 to 0.98; *p* = 0.031 for first HF hospitalization; RR: 0.67, 95% CI 0.51 to 0.89; *p* = 0.006 for total HF hospitalizations). No differences were found in terms of death from any cause [136].

In the DAPA-MI trial, 4017 patients with a recent MI but without known DM or chronic HF were randomized to receive dapagliflozin 10 mg daily or placebo. Over approximately one year of follow-up, dapagliflozin significantly improved cardiometabolic outcomes compared with placebo. The primary hierarchical composite endpoint, which included death, HF hospitalization, nonfatal MI, atrial fibrillation or flutter, new-onset T2DM, change in NYHA class, and ≥5% weight loss, favored dapagliflozin (win ratio 1.34; 95% CI 1.20 to 1.50; *p* < 0.001), mainly driven by the cardiometabolic outcomes. Indeed, the conventional composite of cardiovascular death or HF hospitalization was not significantly different between the two groups, occurring in 2.5% of patients in the dapagliflozin group and 2.6% in the placebo group (HR 0.95; 95% CI 0.64 to 1.40). No new safety signals were identified [137].

In summary, SGLT2-i have demonstrated CV benefits in patients with known ASCVD and T2DM. However, a clear benefit in patients with recent ACS is not confirmed. SGLT2-i are generally well tolerated, with an adverse event profile primarily characterized by an increased risk of urinary tract infections, genital mycotic infections, and volume depletion. Notably, no increased risk of hypoglycemia has been observed [133,138,139,140,141]. Importantly, there is no need to delay the initiation of SGLT2-i in potential patients at the time of hospital discharge [142].

According to the most recent ESC guidelines on CCS, SGLT2-i with proven CV benefit (canaglifozin, dapagliflozin, empagliflozin, sotagliflozin) are recommended in patients with T2DM and CCS, thus in secondary prevention, independently of baseline or target HbA1c and of concomitant glucose-lowering medication, in order to reduce CV events with a class of recommendation I, level of evidence A [3].

Several randomized trials have demonstrated CV benefits of these drugs in patients with HF and CKD, regardless of diabetes status [143,144,145,146,147,148,149]. However, specific data on outcomes in the post-PCI population are missing; therefore, these findings, despite being important and informative, fall outside the primary scope of this review.

### 3.2. Obesity

The cornerstone of obesity treatment is lifestyle modification with a class of recommendation I, level of evidence A [92]. A plant-based diet with an appropriate balance of macro- and micro-nutrients, aimed at reducing ASCVD risk and ensuring controlled energy intake, represents the most effective strategy for weight reduction. When combined with regular physical activity, such dietary measures improve the metabolic profile and overall CVD risk [92].

However, if the results of lifestyle interventions are not satisfactory, the addition of pharmacologic therapy is considered reasonable [150]. Although dedicated randomized trials specifically addressing obesity-targeted pharmacotherapy in obese patients after PCI remain limited, the available data suggest that the favorable effects on systemic inflammation, metabolic dysfunction, and atherosclerotic disease progression may reasonably extend to obese individuals with elevated residual cardiovascular risk following PCI [151].

GLP-1RAs are medications primarily thought to treat T2DM and obesity. Moreover, GLP-1 receptor agonists have demonstrated CV protective effects (Table 3) [152].

Cagrilintide, an amylin analog, is currently under development in combination with the GLP-1 receptor agonist semaglutide to promote durable weight loss in individuals with overweight or obesity [153].

Other drugs available for weight management include orlistat, naltrexone/bupropione, tirzepatide, and setmelanotide. However, these agents are not specifically indicated for secondary CV prevention. Orlistat acts by selectively inhibiting gastric and pancreatic lipases in the intestinal lumen, thereby reducing dietary fat absorption [154], but there are no data on patients with CVD. Naltrexone/bupropione combines naltrexone, an opioid receptor antagonist, with bupropione, a dopamine and norepinephrine reuptake inhibitor. Together, they synergistically stimulate central proopiomelanocortin secretion, leading to reduced food craving and increased satiety [155]. The CV effects are not well defined, and the combined medication is actually under investigation [6].

#### 3.2.1. Glucagon-like Peptide 1 Receptor Agonists for Obesity

GLP-1RAs modulate pancreatic β-cells’ secretion of insulin and, in this way, they regulate glucose homeostasis. An interesting effect of GLP-1RAs concerns the action on stomach and bowel motility. Indeed, GLP-1RAs delay gastric emptying, decrease intestinal motility, and reduce appetite [71]. Intuitively, GLP-1RAs help obese patients control their caloric intake by reducing the feeling of satiety.

Among GLP-1RAs, the European Medicines Agency (EMA) and the Food and Drug Administration (FDA) approved semaglutide and liraglutide in obese patients, both adults and children of ≥12 years of age, in addition to lifestyle modifications [6].

Regarding liraglutide, no CV outcomes trials have been conducted specifically in patients with overweight or obesity, regardless of T2DM (Table 4). However, the SCALE (effect of liraglutide on long-term weight maintenance and additional weight loss induced by a 4 to 12 week low calorie diet in obese subjects; a 56 week randomized, double-blind, placebo controlled, parallel group, multicenter trial with a 12 week follow-up period) program evaluated the effect on weight loss by liraglutide 3 mg administered subcutaneously once daily versus placebo in patients with obesity [156,157,158,159,160]. In these trials, patients with established ASCVD were either excluded or represented in a small proportion (8.5% to 15%) of the study population [157,158]. These randomized controlled trials demonstrated that liraglutide was more effective than placebo in promoting weight loss, whether added to lifestyle modifications or compared with physical exercise or placebo alone [157,158,159,161]. Regarding CV outcomes, in patients with T2DM treated with liraglutide 1.8 mg subcutaneously once daily, MACE and CV death were reduced by 13% and 22%, respectively, compared to placebo [74].

Concerning semaglutide, the STEP (Once-Weekly Semaglutide in Adults with Overweight or Obesity) program evaluated its efficacy and safety in obesity patients with a medium BMI of 37.9 kg/m^2^.

The STEP 1–5 trial program consistently demonstrated that once-weekly subcutaneous semaglutide 2.4 mg induces substantial, clinically meaningful, and durable weight loss in adults with overweight or obesity, including those with type 2 diabetes (STEP 2 trial). Across the studies, semaglutide led to an average body weight reduction ranging from approximately 15% to over 20% at 68 weeks, significantly outperforming placebo and lower-dose (1.0 mg) semaglutide, and showing efficacy comparable to bariatric surgery in selected populations. The addition of intensive behavioral therapy further enhanced weight loss, while treatment discontinuation resulted in weight regain, underscoring the need for long-term therapy. Importantly, weight reduction was sustained for up to 2 years with continued treatment and was accompanied by favorable effects on cardiometabolic parameters, including waist circumference, blood pressure, and lipid profile, supporting the role of semaglutide as an effective pharmacological strategy for sustained weight management [162,163,164,165,166].

Overall, an increased rate of gastrointestinal adverse events was observed with semaglutide compared with placebo, specifically nausea, diarrhea, vomiting, constipation, dyspepsia, and abdominal pain. However, these adverse events were mild to moderate. Usually, these symptoms lasted less than 2 months. On the other hand, semaglutide showed a significantly lower rate of CV events compared to placebo [167].

In STEP 8 (effect and safety of subcutaneous semaglutide 2.4 mg once weekly compared to liraglutide 3.0 mg once daily on weight management in subjects with overweight or obesity) were compared semaglutide 2.4 mg subcutaneously once weekly to liraglutide 3.0 mg subcutaneously daily added to counseling for diet and physical activity. Lower rates of treatment discontinuation for any cause were observed with semaglutide compared with liraglutide, with a similar safety profile. Thus, semaglutide showed significantly greater weight loss at 68 weeks (significantly greater odds of achieving ≥ 10%, ≥15% and ≥20% weight loss with semaglutide vs. liraglutide: 70.9% of participants vs. 25.6%, OR 6.3, 95% CI, 3.5 to 11.2; 55.6% vs. 12.0% OR, 7.9, 95% CI, 4.1 to 15.4 and 38.5% vs. 6.0%, OR, 8.2, 95% CI, 3.5 to 19.1, respectively; all *p* < 0.001) [168].

The SELECT (semaglutide effects on CV outcomes in people with overweight or obesity) trial was the first to evaluate the CV effects of semaglutide in obese patients without diabetes. This multicenter, double-blind, randomized, placebo-controlled, event-driven superiority trial enrolled 17,064 patients aged >45 years with established CVD and a BMI ≥ 27 kg/m^2^ but no history of DM. Treatment with semaglutide 2.4 mg administered subcutaneously once weekly was superior to placebo in reducing the composite outcome of CV death, nonfatal MI, or nonfatal stroke over a mean follow-up of 39.8 months (6.5% vs. 8.0%; HR 0.80; 95% CI 0.72–0.90; *p* < 0.001). The incidence and nature of adverse events were consistent with those observed in previous semaglutide trials [169].

In the OASIS-4 (oral semaglutide at a dose of 25 mg in adults with overweight or obesity) trial oral semaglutide (25 mg) once daily was investigated as an alternative to injected semaglutide in patients with overweight or obesity (BMI ≥ 30 or BMI ≥ 27 with at least one obesity-related complication). Patients were randomized to receive oral semaglutide or placebo, adding lifestyle modifications. Oral semaglutide produced a significantly greater mean reduction in body weight compared with placebo (−13.6% vs. −2.2%), corresponding to an estimated difference of −11.4 percentage points (95% CI −13.9 to −9.0; *p* < 0.001). Participants receiving oral semaglutide were also significantly more likely than those receiving placebo to achieve body-weight reductions of ≥5%, ≥10%, ≥15%, or ≥20% (*p* < 0.001 for all comparisons) and to show improvement in the IWQOL-Lite-CT (impact of weight on quality of life–lite clinical trials version) physical function score (*p* < 0.001). The 25 mg oral formulation achieved weight loss comparable to that observed with higher doses and, in some measures, to injectable formulations [170]. The safety profile was consistent with GLP1-RAs class effects, including mainly gastrointestinal events. An efficacious oral GLP-1 offers practical advantages for adherence and broadening access, potentially useful in the post-PCI population, where the injectable route may be a barrier.

The REDEFINE-1 (coadministered cagrilintide and semaglutide in adults with overweight or obesity) trial is a phase 3, 68-week, multicenter, double-blind, placebo-controlled, and active-controlled trial that enrolled 3417 adults without DM who had a BMI ≥ 30 or a BMI ≥ 27 with at least one obesity-related complication. Participants were randomized to receive cangrisema (2.4 mg + 2.4 mg weekly) versus comparator arms (semaglutide alone, cagrilintide alone, placebo) plus lifestyle counseling over 68 weeks. The cangrisema achieved a mean weight reduction of about −22.7% at 68 weeks in the treatment regimen versus about −2.3% in the placebo. An estimated treatment difference reported was −20.4 percentage points (95% CI, −21.1 to −19.7; *p* < 0.001) under an adherence-assumed analysis; a supportive estimand showed a between-group difference of −17.3 percentage points (95% CI, −18.1 to −16.6; *p* < 0.001) when analyzed regardless of adherence. Combination therapy yielded greater weight loss. An expected increase in gastrointestinal adverse events was observed [171].

Finally, the positive results of the TRIUMPH-4 trial have been announced. The study assessed the efficacy and safety of retatrutide, a once-weekly triple hormone receptor agonist (targeting GLP-1, GIP, and glucagon). In adults with obesity or overweight and knee osteoarthritis, the highest dose of retatrutide led to an average weight loss of 28.7% over 68 weeks. All primary and key secondary endpoints were met, and additional phase 3 trials are expected to report results in 2026. Retatrutide is still investigational and not yet approved [172].

For secondary prevention after PCI, this class of combination agents is promising to markedly reduce adiposity and improve metabolic risk, but CV outcome data in patients with recent ischemia are still needed. In fact, considering that monotherapies with cagrilintide and semaglutide were associated with weight loss [171], their combination (CangriSema) therapy for weight management in adults with T2DM was analyzed in the REDEFINE-2 (cagrilintide-semaglutide in adults with overweight or obesity and T2DM) trial. A total of 1206 adults with BMI ≥ 27, a HbA1c level of 7 to 10%, and T2DM were randomly assigned in a 3:1 ratio to receive once weekly cagrilintide-semaglutide (2.4 mg each) or placebo, along with lifestyle intervention, for 68 weeks. The estimated mean change in body weight from baseline to week 68 was −13.7% in the cangrisema group and −3.4% in the placebo group (estimated difference, −10.4 percentage points; 95% CI, −11.2 to −9.5; *p* < 0.001). More patients in the cangrisema group than in the placebo group had a ≥5% weight reduction (*p* < 0.001). Thus, in populations with T2DM, cangrisema demonstrated significant mean weight reductions (reported ~15.7% in some analyses for patients with T2DM at 68 weeks) and robust improvements in HbA1c versus placebo (between-group differences statistically significant; *p* < 0.001) [173]. Secondary prevention in patients with T2DM, combined incretin/amylin strategies offer a dual metabolic benefit, controlling weight and glycemic levels that may translate to improvement in residual CV risk. Targeted trials in coronary populations are warranted.

According to the most recent ESC guidelines, GLP-1RAs should be considered in patients with T2DM who are overweight or obese to promote weight reduction (Class IIa, Level of Evidence B) [92]. In addition, GLP-1RAs with proven CV benefit, such as dulaglutide, efpeglenatide, liraglutide, and semaglutide, are recommended for patients with T2DM and established ASCVD (Class I, Level of Evidence A) [92]. Moreover, semaglutide should be considered in overweight (BMI > 27 kg/m^2^) or obese patients with CCS, regardless of diabetes status, to reduce the risk of cardiovascular mortality, myocardial infarction, or stroke (Class IIa, Level of Evidence B) [3].

Considering weight loss is fundamental even in T2DM treatment, GLP-1RAs should be considered in patients with obesity or overweight and T2DM to manage BMI with a class of recommendation IIa, level of evidence B [92].

#### 3.2.2. Non-Peptide Glucagon-like Peptide 1 Receptor Agonists for Obesity

Orforglipron (LY3502970) is a novel, oral non-peptide GLP-1RA. In a Phase 1, blinded, placebo-controlled, randomized trial involving healthy participants orforglipron was administered once daily without food or water restrictions. orforglipron demonstrated a substantial reduction in body weight of up to 5.4 kg after 4 weeks compared to a reduction of 2.4 kg in patients treated with placebo (*p* < 0.05). orforglipron decreased fasting glucose levels and gastric emptying. orforglipron had a pharmacodynamic and safety profile similar to that of injectable GLP-1RAs [174].

In the ATTAIN-1 (a study of orforglipron in adult participants with obesity or overweight with weight-related comorbidities) trial, the safety and efficacy of once-daily orforglipron at doses of 6 mg, 12 mg, or 36 mg were compared with placebo on top of a healthy diet and physical activity for 72 weeks. A total of 3127 adults with obesity and without T2DM were enrolled. The body weight mean change from baseline to week 72 was −7.5% (95% CI, −8.2 to −6.8) with 6 mg, −8.4% (95% CI, −9.1 to −7.7) with 12 mg, and −11.2% (95% CI, −12.0 to −10.4) with 36 mg of orforglipron, as compared with −2.1% (95% CI, −2.8 to −1.4) with placebo (*p* < 0.001, for all comparisons with placebo). Among the patients in the orforglipron 36 mg group, 54.6% had a reduction of ≥10%, 36.0% had a reduction of ≥15%, and 18.4% had a reduction of ≥20% or more, as compared with 12.9%, 5.9%, and 2.8% of the patients, respectively, in the placebo group. A significant improvement of waist circumference, SBP, triglycerides, cholesterol (TG) levels, and non-high-density lipoprotein (HDL) cholesterol levels was observed in the orforglipron group compared to placebo. Adverse events resulted in treatment discontinuation in 5.3 to 10.3% of the patients in the orforglipron groups and in 2.7% of those in the placebo group, respectively. The most common adverse events with orforglipron were mild to moderate gastrointestinal effects. However, the mean percent weight loss was generally numerically lower than the top-tier peptide injectables (e.g., tirzepatide at maximal doses) [175]. Oral small-molecule GLP-1 agonists can increase accessibility to this drug class. However, when the clinical objective is maximal adiposity reduction (such as in very-high-risk post-PCI patients), peptide-based or dual-agonist strategies may be preferred pending CV outcome data.

#### 3.2.3. Glucagon-like Peptide 1 Receptor Agonists and Glucose-Dependent Insulinotropic Peptide for Obesity

Tirzepatide was investigated in overweight and obese patients in the SURMOUNT-1 (efficacy and safety of tirzepatide once weekly in participants without Type 2 Diabetes who have obesity or are overweight with weight-related comorbidities: a randomized, double-blind, placebo-controlled trial) trial. Tirzepatide demonstrated a dose-dependent body weight reduction (mean percentage change in weight at week 72: −15.0%, 95% CI, −15.9 to −14.2 with 5 mg weekly doses of tirzepatide; −19.5%, 95% CI, −20.4 to −18.5, with 10 mg doses; −20.9%, 95% CI, −21.8 to −19.9 with 15 mg doses; −3.1%, 95% CI, −4.3 to −1.9 with placebo, *p* < 0.001 for all comparisons with placebo) [176]. Subsequently, tirzepatide was compared with semaglutide 1.0 mg subcutaneously (but not with the 2.4 mg dose) in the SURPASS-2 trial, demonstrating non-inferiority and superiority in weight loss and HbA1c-lowering values in obese patients with T2DM [97]. Recent findings showed that, after three years of treatment with tirzepatide, obese patients with prediabetes experienced sustained weight loss, along with a significantly lower risk of progression to T2DM compared to placebo [177]. Actually, SURMOUNT-MMO (a study of tirzepatide on the reduction inof morbidity and mortality in adults with obesity) trial (NCT05556512) is ongoing to evaluate the effect of tirzepatide on CV outcomes in adults with obesity without T2DM [6]. The recent SURMOUNT-5 (tirzepatide as compared with semaglutide for the treatment of obesity) trial directly compared tirzepatide and semaglutide in adults with obesity but without T2DM. This phase 3b, open-label, randomized controlled trial enrolled 751 participants who were randomized 1:1 to receive the maximum tolerated dose of tirzepatide (10 or 15 mg) or semaglutide (1.7 or 2.4 mg) once weekly for 72 weeks (modified treatment-regimen estimand). The primary endpoint was the percent change in body weight from baseline to week 72. Tirzepatide achieved a least-squares mean percent reduction of −20.2% (95% CI −21.4 to −19.1) compared with −13.7% (95% CI −14.9 to −12.6) with semaglutide, corresponding to an estimated treatment difference of −6.5 percentage points (95% CI −8.1 to −4.9; *p* < 0.001). Tirzepatide also produced greater reductions in waist circumference (−18.4 cm vs. −13.0 cm; difference −5.4 cm; 95% CI −7.1 to −3.6; *p* < 0.001) and higher proportions of participants achieving categorical weight-loss thresholds (≥20%: 48.4% vs. 27.3%). Mild-to-moderate gastrointestinal adverse events were the most common, while the incidence of serious adverse events was similar between groups [178]. Although SURMOUNT-5 enrolled participants without recent ischemic events, the magnitude of weight loss and improvements in cardiometabolic risk factors make tirzepatide highly relevant for consideration in high-risk settings such as post-PCI patients with obesity; however, direct evidence on MACE reduction post-PCI is lacking. Intriguingly, an observational study of CV Outcomes of Tirzepatide versus GLP1-RAs examined CV benefits of tirzepatide compared to GLP-1RAs in people ≥ 40 years old with overweight or obesity affected by T2DM and pre-existing CAD. Tirzepatide treatment was related both with a lower primary composite outcomes (acute MI, ischemic stroke and all-cause mortality compared to GLP-1RAs (HR: 0.60, 95% CI: 0.43–0.84, *p* < 0.001) and lower rate of acute myocardial infarction (HR: 0.59, 95% CI: 0.38 to 0.91) and all-cause mortality (HR: 0.35, 95% CI: 0.14 to 0.88, *p* = 0.001) alone [179]. More randomized trials pointing out CV outcomes are needed. Tirzepatide showed CV benefits also in HF patients. In the SUMMIT (a randomized, double-blind, placebo-controlled, Phase 3 study comparing the efficacy and safety of tirzepatide versus placebo in patients with heart failure with preserved ejection fraction and obesity) trial, tirzepatide was evaluated in 731 patients with heart failure with preserved ejection fraction (HFpEF) and obesity (BMI ≥ 30 kg/m^2^). Over approximately 104 weeks, tirzepatide significantly reduced death and worsening heart failure events compared with placebo [180]. Although HFpEF differs from post-MI or post-PCI ischemic disease, these findings support the potential of intensive metabolic therapy targeting adiposity to improve cardiovascular outcomes [181]. The 2025 ACC scientific statement outlines optimal approaches for diagnosing, evaluating, and managing obesity in adults with heart failure, emphasizing lifestyle changes, pharmacotherapy (including emerging agents like semaglutide and tirzepatide), and surgical options while highlighting knowledge gaps and challenges such as appropriate obesity metrics and safety in particular in patients with heart failure with reduced ejection fraction (HFrEF) [182]. However, these trials focused on obesity and HF do not specifically address post-PCI outcomes. Thus, these findings lie outside the scope of this review.

**Table 1:** Main randomized trials of treatment with GIP and GLP-1 agonist with diabetes and CVD.

	Patients (*n*)	Year of Publication	Population Characteristics	Randomized Arms	Primary Endpoint	Main Results
ELIXA Trial [74]	6068	2015	Patients with T2DM with a previous MI or who had been hospitalized for unstable angina	To receive lixisenatide plus OMT or placebo plus OMT	A composite of CV death, MI, stroke, or hospitalization for unstable angina	Lixisenatide showed noninferiority but not superiority to placebo (13.4% vs. 13.2%, HR: 1.02; 95% CI, 0.89 to 1.17; *p* < 0.001 for noninferiority, *p* = 0.81 for superiority)
LEADER Trial [73]	9340	2016	Patients with T2DM and high CV risk	To receive liraglutide or placebo	A composite of CV death, nonfatal MI or nonfatal stroke	Liraglutide was associated with a significant reduction in primay endpoint compared with placebo (13.0% vs. 14.9%, HR: 0.87; 95% CI, 0.78 to 0.97; <0.001 for noninferiority, *p* = 0.01 for superiority)
EXSCEL Trial [78]	14,752	2017	Patients with T2DM, with or without previous CV disease	To receive subcutaneous injection of 2 mg exenatide or matching placebo once weekly	A composite of the first occurrence of death from CV causes, nonfatal MI, or nonfatal stroke	Exenatide showed noninferiority with respect to safety, but not superiority to placebo with respect to efficacy (11.4% vs. 12.2%, HR: 0.91; 95% CI, 0.83 to 1.00; *p* < 0.001 for noninferiority, *p* = 0.06 for superiority)
HARMONY Outcome Trial [75]	9463	2018	Patients > 40 years of age with T2DM and established ASCVD	To receive subcutaneous injection of albiglutide or matching placebo	A composite of CV death, MI, or stroke	Albiglutide was associated with a significant reduction in primary endpoint compared with placebo (7.0% vs. 9.0%, HR: 0.78; 95% CI, 0.68 to 0.90; *p* < 0.0001 for non-inferiority; *p* = 0.0006 for superiority).
REWIND Trial [76]	9901	2019	Patients ≥ 50 years with T2DM who had either a previous CV event or CV risk factors	To receive weekly subcutaneous injection of dulaglutide 1.5 mg or placebo	A composite of the first occurrence of non-fatal MI, non-fatal stroke, or death from CV causes	Dulaglutdie was associated with a significant reduction in primary endpoint compared with placebo (12.0% vs. 13.4%, HR: 0.88; 95% CI, 0.79 to 0.99; *p* = 0.026)
AMPLITUDE-O Trial [79]	4076	2021	Patients with T2DM and either a history of CV disease or current kidney disease	To receive weekly subcutaneous injections of efpeglenatide at a dose of 4 or 6 mg or placebo	A composite of nonfatal MI, nonfatal stroke, or death from CV or undetermined causes	Efpeglenatide was associated with a significant reduction in primary endpoint compared with placebo (7.0% vs. 9.2%, HR: 0.73; 95% CI, 0.58 to 0.92; *p* < 0.001 for noninferiority; *p* = 0.007 for superiority)
SUSTAIN-6 Trial [81]	3297	2016	Patients with T2DM	To receive once-weekly semaglutide (0.5 mg or 1.0 mg) or placebo	A composite of first occurrence of CV death, nonfatal MI or nonfatal stroke	Semaglutide was associated with a significant reduction in primary endpoint compared with placebo (6.6% vs. 8.9%, HR: 0.74; 95% CI, 0.58 to 0.95; *p* < 0.001 for noninferiority)
PIONEER 1 Trial [82]	703	2019	Patient with T2DM insufficiently controlled with diet and exercise	To receive once-daily oral semaglutide 3 mg, 7 mg, 14 mg or placebo	Change from baseline to week 26 in HbA1c	Semaglutide demonstrated superior and clinically relevant improvements in HbA1c (−0.6%, −0.9%, −1.1%; *p* < 0.001 for all comparisons)
PIONEER 2 Trial [88]	787	2019	Patient with T2DM uncontrolled on metformin	To receive oral semaglutide 14 mg or empaglifozin 25 mg	Change from baseline to week 26 in HbA1c	Semaglutide was superior to empagliflozin in reducing HbA1c (−1.3% vs. −0.9%, ETD −0.4% [95% CI, −0.6 to 0.3] −5 mmol/mol; *p* < 0.0001)
PIONEER 6 Trial [83]	3183	2019	Patients at high CV risk (age ≥ 50 years with established CV or chronic kidney disease, or ≥60 years with CV risk factors only)	To receive oral semaglutide or placebo	A composite of CV death, nonfatal MI or nonfatal stroke	Semaglutide was associated with a significant reduction in primary endpoint compared with placebo (3.8% vs. 4.8%, HR: 0.79; 95% CI, 0.57 to 1.11; *p* < 0.001)
FLOW Trial [85]	3533	2024	Patients with T2DM and chronic kidney disease	To receive 1 mg of subcutaneous semaglutide once weekly or placebo	Major kidney disease events, a composite of the onset of kidney failure, at least a 50% reduction in the eGFR from baseline, or death from kidney-related or CV causes	The risk of a primary-outcome event was 24% lower in the semaglutide group than in the placebo group (331 vs. 410 first events, HR: 0.76; 95% CI, 0.66 to 0.88; *p* = 0.0003)
STRIDE Trial [87]	792	2025	Patients with T2DM and PAD with intermittent claudication	To receive 1 mg of subcutaneous semaglutide or placebo once week	The ratio to baseline of the maximum walking distance at week 52	Semaglutide was superior to placebo (estimated median ratio of 1.21 (IQR 0.95 to 1.55) vs. 1.08 (IQR 0.86 to 1.36); estimated treatment ratio 1.13 (95% CI, 1.06 to 1.21, *p* = 0.0004)
SOUL Trial [86]	9650	2025	Patients ≥ 50 years with T2DM with HbA1c level of 6.5 to 10% and known ASCVD, CKD or both	To receive once-daily oral semaglutide (maximal dose, 14 mg) plus OMT or placebo plus OMT	A composite of death from CV causes, nonfatal MI or nonfatal stroke	Semaglutide was associated with a significantly lower risk of major adverse cardiovascular events than placebo (12.0% vs. 13.8%, HR: 0.86; 95% CI, 0.77 to 0.96, *p* = 0.006)
SURPASS-4 Trial [101]	2002	2021	Patients had T2DM treated with any combination of metformin, sulfonylurea, or SGLT2-i, a baseline glycated hemoglobin of 7.5–10.5%, BMI ≥ 25, and established cardiovascular disease or a high risk of CV events	To receive tirzepatide at a dose of 5 mg, 10 mg, or 15 mg, or glargine 100 U/mL	The non-inferiority (0.3% non-inferiority boundary) of tirzepatide 10 mg or 15 mg, or both, versus glargine in HbA1c change from baseline to 52 weeks	Tirzepartide was superior to glargine (−2.43 with 10 mg, −2.58% with 15 mg vs. −1.14% with glargine (ETD: −0.99%; 97.5% CI, −1.13 to −0.86 with 10 mg and −1.14% 97.5% CI, −1.28 to −1.00 with 15 mg; non-inferiority of 0.3% met for both doses)
SURPASS-4 Trial substudy [102]	2002	2022	Patients had T2DM treated with any combination of metformin, sulfonylurea, or SGLT2-i, a baseline glycated hemoglobin of 7.5–10.5%, BMI ≥ 25, and established CV disease or a high risk of CV events	To receive tirzepatide at a dose of 5 mg, 10 mg, or 15 mg, or glargine 100 U/mL	Time to first occurrence of eGFR decline of at least 40% from baseline, end-stage kidney disease, death owing to kidney failure, or new-onset macroalbuminuria	Tirzepatide slowed the rate of eGFR decline and reduced UACR in clinically meaningful ways compared with insulin glargine (mean rate of eGFR decline −1.4 mL/min/1.73 m^2^ per year vs. −3.6 mL/min/1.73 m^2^ per year; between group difference of 2.2; CI 95%, 1.6 to 2.8)

ASCVD: atherosclerotic cardiovascular disease, BMI: body mass index, CI: confidence interval, CV: cardiovascular, eGFR: estimated glomerular filtration rate, ETD: estimated treatment difference, GLP-1: glucagon like peptide-1, HR: hazard ratio, MI: myocardial infarction, OMT: optical medical therapy, PAD: peripheral artery disease, SGLT2-i: sodium-glucose cotransporter-2 inhibitors, T2DM: type 2 diabetes mellitus.

**Table 2:** Main randomized trials of treatment with SGLT2-i.

	Patients (*n*)	Year of Publication	Population Characteristics	Randomized Arms	Primary Endpoint	Main Results
EMPA-REG OUTCOME Trial [115]	7020	2015	Patients with T2DM at high CV risk	To receive empagliflozin 10 mg or 25 mg or placebo	A composite of death from CV causes, nonfatal MI or nonfatal stroke	Empagliflozin was superior to placebo (10.5% vs. 12.1%, HR: 0.86; 95% CI, 0.74 to 0.99; *p* = 0.04)
EMPEROR-Reduced Trial [138]	3730	2020	Patients with class II to IV HF with an EF ≤ 40%	To receive empagliflozin 10 mg or placebo, both in addition to OMT	A composite of CV death or hospitalization for HF	Empagliflozin was superior to placebo (19.4% vs. 24.7%, HR: 0.75; 95% CI, 0.65 to 0.86; *p* < 0.0001
EMMY trial [131]	476	2022	Patients with acute MI accompanied by a large creatine kinase elevation (>800 IU/L)	To receive empagliflozin 10 mg or placebo	NT-proBNP change over 26 weeks	Empagliflozin was associated with a significantly greater NT-proBNP reduction (15% lower, 95% CI, −4.4% to −23.6%; *p* = 0.026)
EMPACT-MI trial [132]	6522	2024	Patients hospitalized for acute MI and at risk for HF	To receive empagliflozin 10 mg plus OMT or placebo plus OMT	A composite of hospitalization for HF or death from any cause	Empagliflozin was not superior to placebo (8.2% vs. 9.1%, HR: 0.90; 95% CI, 0.76 to 1.06; *p* = 0.21)
DECLARE-TIMI 58 trial [117]	17,160	2019	Patients with T2DM and ASCVD or multiple risk factor	To receive dapagliflozin or placebo	A composite of CV death, MI, ischemic stroke or hospitalization for HF	Dapagliflozin did not change MACE than placebo (8.8% vs. 9.4%, HR: 0.93; 95% CI, 0.84 to 1.03; *p* = 0.17) but significantly reduced cardiovascular death or hospitalization for heart failure (4.9% vs. 5.8%, HR: 0.83; 95% CI, 0.73 to 0.95; *p* = 0.005)
DAPA-HF trial [140]	4744	2019	Patients with class II to IV HF with an EF ≤ 40%	To receive dapagliflozin 10 mg plus OMT vs. placebo plus OMT	A composite of worsening HF or CV death	Dapagliflozin significantly reduced the primary endpoint compared with placebo (16.3% vs. 21.2%, HR: 0.74; 95% CI, 0.65 to 0.85; *p* < 0.001)
DAPA-CKD trial [146]	4304	2020	Patients with eGFR of 25 to 75 and albumin/creatinine ratio of 200 to 5000	To receive dapagliflozin 10 mg or placebo	A composite of a sustained decline of eGFR, end stage kidney disease, or renal or CV death	Dapagliflozin significantly reduced the primary endpoint compared with placebo (9.2% vs. 14.5%, HR: 0.61; 95% CI, 0.51 to 0.72; *p* < 0.001)
DAPA-MI trial [134]	4017	2024	Patients with acute MI and impaired LVS function, without prior diabetes or chronic HF	To receive dapagliflozin 10 mg or placebo	A composite of death, hospitalization for HF, nonfatal MI, atrial fibrillation/flutter, T2DM, NYHA at last visit and body weight decrease of ≥5% at last visit	Dapagliflozin led to significant benefits in terms of cardiometabolic outcomes compared with placebo (Win ratio: 1.34; 95% CI, 1.20 to 1.50; *p* < 0.001)
CANVAS and CANVAS-R trial [121]	10,142	2017	Patients with T2DM and high CV risk	To receive canagliflozin 100 mg or placebo	A composite of death from CV causes, nonfatal MI or nonfatal stroke	Canagliflozin significantly reduced CV events compared with placebo (26.9 vs. 31.5 participants per 1000 patient-years, HR: 0.86; 95% CI; 0.75 to 0.97; *p* < 0.001 for noninferiority, *p* = 0.02 for superiority)
CANVAS and CANVAS-R trial Substudy [122]	10,142	2018	Patients with T2DM and high CV risk	To receive canagliflozin 100 mg or placebo	A composite of sustained and adjudicated doubling in serum creatinine, end-stage kidney disease or death for renal causes	Canagliflozin treatment was associated with a reduced risk of sustained loss of kidney function (1.5 vs. 2.8 participants per 1000 patient-years, HR: 0.53; 95% CI, 0.33 to 0.84)
CREDENCE trial [125]	4401	2019	Patients with T2DM and albuminuric CKD	To receive canagliflozin 100 mg or placebo	A composite of end-stage kidney disease, a doubling of the serum creatinine or death for CV or renal causes	Canagliflozin significantly reduced the risk of kidney failure and cardiovascular events compared with placebo (43.2 vs. 61.2 participants per 1000 patient-years, HR: 0.70; 95% CI, 0.59 to 0.82; *p* = 0.00001)
VERTIS CV trial [129]	8246	2020	Patients with T2DM and ASCVD	To receive 5 mg or 15 mg of ertugliflozin or placebo	A composite of death from CV causes, nonfatal MI or nonfatal stroke	Ertugliflozin was non inferior to placebo in terms of major adverse cardiovascular events (11.9% vs. 11.9%, HR: 0.97; 95.6% CI, 0.85 to 1.11; *p* < 0.001 for noninferiority)

ASCVD: atherosclerotic cardiovascular disease, BMI: body mass index, CI: confidence interval, CV: cardiovascular, EF: ejection fraction, eGFR: estimated glomerular filtration rate, HF: heart failure, HR: hazard ratio, LVS: left ventricular systolic, MI: myocardial infarction, NT-proBNP: N-terminal pro-brain natriuretic peptide, NYHA: New York association functional classification, OMT: optical medical therapy, SGLT2-i: sodium-glucose cotransporter-2 inhibitors, T2DM: type 2 diabetes mellitus.

**Table 3:** Main randomized trials of treatment with GIP and GLP-1 agonist for obesity and weight loss in patients with diabetes.

	Patients (*n*)	Year of Publication	Population Characteristics	Randomized Arms	Primary Endpoint	Main Results
SCALE Diabetes trial [155]	846	2015	Patients with BMI ≥ 27 kg/m^2^, taking 0 to 3 oral hypoglycemic agents with stable body weight and HbA1c 7% to 10%	To receive once daily subcutaneous liraglutide 1.8 mg, 3.0 mg or placebo	Relative change in weight at week 56	Weight loss was 2.0% with placebo, 4.7% with liraglutide 1.8 mg (EMD vs. placebo: −2.71% [95% CI, −4.0% to 1.42%]) and 6.0% with liraglutide 3.0 mg (EMD vs. placebo: −4.0% [95% CI, −5.1% to 2.9%]) *p* < 0.001 for both
STEP 2 Trial [159]	1210	2021	Patient with BMI ≥ 27 kg/m^2^ and T2DM with HbA1c 7 to 10%	To receive 2.4 mg subcutaneous semaglutide once a week or placebo	Percentage change in body weight	Semaglutide led to a superior decrease in bodyweight compared with placebo (−9.6% vs. 3.4%, 95% CI, −7.3 to −5.2, *p* < 0.001)
REDEFINE 2 trial [169]	1206	2025	Patient with BMI ≥ 27 kg/m^2^ and T2DM with HbA1c 7 to 10%	To receive once-weekly cagrilintide-semaglutide (2.4 mg each) or placebo	Percentage change in body weight	Cagrilintide-semaglutide treatment was associated with a significantly lower body weight than placebo (−13.7% vs. −3.4%, EMD −10.4%, 95% CI 95%, −11.2 to −9.5; *p* < 0.001)
SURPASS-1 trial [93]	478	2021	Patients with T2DM inadequately controlled by diet and exercise alone and if they were naive to injectable diabetes therapy	To receive 5 mg, 10 mg, or 15 mg of tirzepatide or placebo	The mean change in HbA1c from baseline at 40 weeks	Tirzepatide improved glycemic control compared with placebo (EMD vs. placebo of −1.91% with 5 mg, −1.93% with 10 mg, and −2.11% with 15 mg; *p* < 0.0001 for all)
SURPASS-2 trial [96]	1879	2021	Patients with T2DM inadequately controlled with metformin, HbA1c of 7.0 to 10.5% and BMI ≥ 25 kg/m^2^	To receive tirzepatide at a dose of 5 mg, 10 mg, or 15 mg or semaglutide at a dose of 1 mg	The mean change in HbA1c from baseline at 40 weeks	Tirzepatide was noninferior and superior to semaglutide (EMD between the 5 mg, 10 mg, and 15 mg tirzepatide groups and the semaglutide group were −0.15% [95% CI, −0.28 to −0.03, *p* = 0.02], −0.39% [95% CI, −0.51 to −0.26, *p* < 0.001], and −0.45% [95% CI, −0.57 to −0.32; *p* < 0.001], respectively)
SURPASS-3 trial [98]	1444	2021	Patients with T2DM and HbA1c 7.0 to 10.5% and BMI ≥ 25 were insulin-naive and treated with metformin alone or in combination with an SGLT2-i	To receive tirzepatide at a dose of 5 mg, 10 mg, or titrated insulin degludec	The non-inferiority of tirzepatide 10 mg or 15 mg, or both, versus insulin degludec in mean change in HbA1c from baseline at week 52	Tirzepatide was superior to titrated insulin degludec (reduction of 1.34% for insulin degludec vs. 1.93% for tirzepatide 5 mg, 2.20% for 10 mg, and 2.37% for 15 mg; noninferiority margin of 0.3% was met; ETD −0.59% to −1.04%; *p* < 0.0001)
SURPASS-3 trial substudy [100]	296	2022	Patients with T2DM and HbA1c of 7.0 to 10.5% and BMI ≥ 25 kg/m^2^, insulin-naive and treated with metformin alone or in combination with an SGLT2-i	To receive tirzepatide at a dose of 5 mg, 10 mg, or 15 mg or titrated insulin degludec	The change from baseline in liver fat content at week 52 using pooled data from the tirzepatide 10 mg and 15 mg groups versus insulin degludec	Tirzepatide was superior to titrated insulin degludec (−8.09% vs. −3.38%, EMD of −4.71%; 95% CI, −6.72 to −2.70; *p* < 0.0001)
SURPASS-5 trial [103]	475	2022	Patients with T2DM with inadequate glycemic control on once-daily insulin glargine with or without metformin	To receive once-weekly subcutaneous injections of 5 mg, 10 mg or 15 mg tirzepatide or placebo	The mean change from baseline in HbA1c at week 40	Tirzepatide significantly improved glycemic control compared with placebo (−0.86% with placebo, −2.11% with tirzepatide 5 mg [difference −1.24%; 95% CI, −1.48 to −1.01%], −2.40% with 10 mg [difference −1.53%; 97.5% CI, −1.80% to −1.27%], −2.34% with 15 mg [difference −1.47; 97.5% CI, −1.75% to −1.20%] *p* < 0.001 for all comparisons)
SURPASS J-mono trial [105]	636	2022	Patients 20 years or older with T2DM who had discontinued oral antihyperglycaemic monotherapy or were treatment-naïve	To receive once-weekly subcutaneous injections of 5 mg, 10 mg or 15 mg tirzepatide or dulaglutide 0.75 mg	The mean change in HbA1c from baseline at week 52	Tirzepatide was superior compared with dulaglutide for glycemic control (difference of −1.3 with dulaglutide vs. −2.4 with tirzepatide 5 mg [EMD vs. dulaglutide −1.1; 95% CI, −1.3 to −0.9], −2.6 with 10 mg [EMD vs. dulaglutide −1.3; 95% CI, −1.5 to −1.1], and −2.8 with 15 mg [EMD vs. dulaglutide −1.5; 95% CI −1.71 to −1.4]; all *p* < 0.0001)
SURPASS J-mono trial substudy [106]	48	2022	Patients 20 years or older with T2DM who had discontinued oral antihyperglycaemic monotherapy or were treatment-naïve	To receive once-weekly subcutaneous injections of 5 mg, 10 mg or 15 mg tirzepatide or dulaglutide 0.75 mg	Postprandial metabolic variables and appetite after a meal tolerance test	Compared with dulaglutide, tirzepatide showed greater potential for normalizing metabolic factors after a standardized meal
SURPASS J-combo Trial [107]	443	2022	Patients with T2DM who had inadequate glycemic control with stable doses of various oral antihyperglycaemic monotherapies	To receive 5, 10, or 15 mg of tirzepatide plus oral antihyperglycaemic monotherapies	Safety and tolerability during 52 weeks of treatment	Tirzepatide was well tolerated as an add-on to oral antihyperglycaemic monotherapy in Japanese participants with T2DM

BMI: body mass index; CI: confidence interval; EMD: estimated mean difference; GIP: glucose-dependent insulinotropic polypeptide; GLP-1: glucagon like peptide-1; SGLT2-i: sodium-glucose cotransporter-2 inhibitors; T2DM: type 2 diabetes mellitus.

**Table 4:** Main randomized trials of treatment with GIP and GLP-1 agonist for obesity and weight loss in patients without diabetes.

	Patients (*n*)	Year of Publication	Population Characteristics	Randomized Arms	Primary Endpoint	Main Results
SCALE Maintenance trial [153]	422	2013	Patients obese/overweight who lost > 5% of initial weight during a low-calorie diet run-in	To receive 3 mg liraglutide or placebo daily	Percentage weight change from randomization, the proportion of participants that maintained the initial > 5% weight loss, and proportion that lost > 5% of randomization weight	Liraglutide, with diet and exercise, maintained weight loss achieved by caloric restriction and induced further weight loss (−6.2% vs. −0.2%, estimated difference −6.1%; 95% CI, −7.5 to 4.6, *p* < 0.0001)
SCALE obesity and Prediabetes trial [155]	3731	2015	Patient without T2DM and BMI ≥ 30 kg/m^2^ or ≥27 kg/m^2^ if they had dyslipidemia or hypertension	To receive daily subcutaneous liraglutide 3.0 mg or placebo	The change in body weight and the proportions of patients losing at least 5% and more than 10% of their initial body weight	Liraglutide was associated with reduced body weight (body weight loss of 8.4 ± 7.3 kg vs. 2.8 ± 6.5 kg; difference of −5.6 kg, 95% CI −6.0 to 5.1; *p* < 0.001) with a significant higher proportion of patients losing at least 5% (63.2% vs. 27.1%; *p* < 0.001) and more than 10% (33.1% vs. 10.6%; *p* < 0.001) of the initial body weight
SCALE obesity and Prediabetes trial (3 year long study) [6]	2254	2017	Patient with prediabetes and BMI ≥ 30 kg/m^2^ or ≥27 kg/m^2^ if they had comorbidities	To receive daily subcutaneous liraglutide 3.0 mg or placebo	Time to diabetes onset by 160 weeks	Liraglutide reduced risk of diabetes in individuals with obesity and prediabetes (2.7 times longer with liraglutide than with placebo, 95% CI, 1.9 to 3.9; *p* < 0.0001)
SCALE IBT Trial [156]	282	2020	Patients with obesity	To receive liraglutide 3.0 mg plus IBT or placebo plus IBT	Weight loss in individual with obesity	Liraglutide enhanced weight loss of IBT compared with placebo (−7.5% vs. −4.0%; ETD −3.4%, 95% CI, −5.3% to −1.6%, *p* = 0.0003)
S-LITE Trial [157]	195	2021	Patients with obesity and BMI of 32 to 43 kg/m^2^ without diabetes	To receive placebo plus a moderate to vigorous intensity exercise program (exercise group); liraglutide 3.0 mg with usual activity (liraglutide group); liraglutide 3.0 mg with exercise program (combined group); placebo plus usual activity (placebo group)	Change in body weight	A strategy combining exercise and liraglutide therapy improved healthy weight loss maintenance more than either treatment alone (−4.1 kg in the exercise group [95% CI, −7.8 to −0.4; *p* = 0.03], −6.8 kg in the liraglutide group [95% CI, −10.4 to −3.1; *p* < 0.001], −9.5 kg in the combined group [95% CI, −13.1 to −5.9; *p* < 0.001])
STEP 1 trial [158]	1961	2021	Patient with BMI ≥ 30 kg/m^2^ or ≥27 kg/m^2^ if they had comorbidities	To receive subcutaneous semaglutide 2.4 mg or placebo	Percentage change in body weight and weight reduction of at least 5%	Semaglutide was associated with significant reduction in body weight compared with placebo (−14.9% vs. −2.4%, 95% CI, −13.4 to −11.5; *p* < 0.001) with a greater rate of patient with a ≥5% reduction (86.4% vs. 31.5%; *p* < 0.001)
STEP 3 Trial [160]	611	2021	Patients with BMI ≥ 27 kg/m^2^ and comorbidities or obesity (BMI ≥ 30 lg/m^2^)	To receive semaglutide 2.4 mg vs. placebo	Percentage in body weight and the loss of 5% or more of baseline weight by week 68	Semaglutide was associated with significant reduction in body weight compared with placebo (−16.0% vs. −5.7%, differenc of −10.3% [95% CI, −12.0 to −8.6; *p* < 0.001] with a greater rate of patient with a ≥5% reduction (86.6% vs. 47.6%; *p* < 0.001)
STEP 4 trial [161]	803	2021	Patient with BMI ≥ 30 kg/m^2^ or ≥27 kg/m^2^ if they had comorbidities	To receive subcutaneous semaglutide 2.4 mg with switch to placebo for weight maintenance	Percentage change in body weight from week 20 to week 68	Maintaining treatment with semaglutide compared with switching to placebo resulted in continued weight loss (−7.9% vs. + 6.9%; difference of −14.8%, 95% CI, −16.0 to −13.5; *p* < 0.001)
STEP 5 trial [162]	304	2022	Patients with obesity or overweight with at least one weight-related comorbidity without diabetes	To receive semaglutide 2.4 mg once weekly plus behavioral intervention vs. placebo plus behavioral intervention	Percentage in body weight and achievement of weight loss of ≥5% at week 104	Semaglutide treatment led to substantial weight loss compared with placebo (−15.2% vs. −2.6%; estimated difference of −12.6%, 95% CI, −15.3 to −9.8; *p* < 0.0001) with a greater rate of patient with a ≥5% reduction (77.1% vs. 34.4%; *p* < 0.0001)
STEP 8 Trial [164]	338	2022	Patients with obesity (BMI ≥ 30 kg/m^2^) or with BMI ≥ 27 kg/m^2^ with at least one weight-related comorbidity without diabetes	To receive semaglutide 2.4 mg once weekly or matching placebo, liraglutide 3.0 mg once daily or matching placebo	Percentage change in body weight	Semaglutide was associated with significantly greater weight loss compared with liraglutide (−15.8% vs. −6.4%; difference of −9.4%, 95% CI, −12.0 to −6.8; *p* < 0.001)
SELECT trial [165]	17,604	2023	Patients older than 45 years with CV disease and BMI ≥ 27 kg/m^2^ but without history of diabetes	To receive semaglutide 2.4 mg once weekly vs. placebo	A composite of death from CV causes, nonfatal MI or nonfatal stroke	Semaglutide was superior to placebo (6.5% vs. 8.0%, HR: 0.80, 95% CI, 0.72 to 0.90; *p* < 0.001)
REDEFINE-1 trial [167]	3417	2025	Patient with BMI ≥ 30 kg/m^2^ or ≥27 kg/m^2^ if they had at least one obesity-related complication	To receive the combination of semaglutide at a dose of 2.4 mg and cagrilintide at a dose of 2.4 mg, semaglutide alone at a dose of 2.4 mg, cagrilintide alone at a dose of 2.4 mg, or placebo	The relative change in body weight	Cagrilintide–semaglutide provided significant and clinically relevant body-weight reductions compared with placebo (−20.4% with cagrilintide-semaglutide vs. −3.0% with placebo; estimated difference of −17.3%, 95% CI, −18.1 to −16.6; *p* < 0.001)

BMI: body mass index, CI: confidence interval, CV: cardiovascular, ETD: estimated treatment difference, GIP: glucose-dependent insulinotropic polypeptide, GLP-1: glucagon-like peptide-1, HR: hazard ratio, IBT: intensive behavioral therapy.

## 4. Gaps in Knowledge

Despite the growing body of evidence supporting metabolic and obesity-targeted therapies, the currently available data are characterized by some limitations. Most clinical trials have enrolled heterogeneous populations with respect to cardiovascular risk, baseline metabolic status, regardless of ASCVD, while using diverse endpoints from weight reduction to surrogate cardiometabolic markers rather than CV outcomes. Moreover, patients undergoing PCI are largely underrepresented or analyzed only as subgroups, limiting the generalizability of trial findings. As a result, significant knowledge gaps remain regarding the optimal selection, timing, and long-term impact of metabolic interventions on residual cardiovascular risk, plaque progression, inflammation, and clinical outcomes after PCI. Dedicated, adequately powered randomized studies focusing on post-PCI populations are therefore warranted to clarify the role of metabolic-targeted therapies individually and in the context of all pharmacotherapies and lifestyle interventions for secondary prevention.

## 5. Conclusions

Patients undergoing PCI for acute or chronic coronary syndrome remain at very high CV risk over time, highlighting the need for structured and individualized secondary prevention strategies to mitigate MACE risk beyond revascularization alone, including OMT and lifestyle interventions. Diabetes and obesity are key modifiable conditions, and their targeted treatment represents a clinically actionable opportunity to reduce residual cardiovascular risk in daily practice. Medical therapies such as SGLT2-i and GLP-1RAs may have a crucial impact on CV outcomes, not only through metabolic control but also via pleiotropic effects on inflammation, atherosclerotic disease progression, and vascular function. Semaglutide, in particular, has been shown to reduce CV events in obese patients, regardless of diabetes status, and should be considered as part of an integrated secondary prevention strategy in patients with CCS and BMI > 27 kg/m^2^, while tirzepatide is currently under investigation for cardiovascular outcomes in obesity without diabetes. In patients with T2DM and excess weight undergoing PCI, weight-lowering glucose-lowering agents, especially GLP-1 RAs, should be prioritized to simultaneously address metabolic control and cardiovascular risk reduction. Overall, contemporary PCI management should move toward a comprehensive approach that systematically incorporates assessment and treatment of residual risk, including strict control of diabetes, obesity, dyslipidemia, and inflammation, in order to prevent recurrent ischemic events and improve long-term outcomes. New evidence in this specific setting of patients is; however, warranted in the near future.

## Data Availability

All data underlying this article will be shared on reasonable request to the corresponding author.

## Abbreviations

The following abbreviations are used in this manuscript:

ACS	Acute Coronary Syndrome
AGE	Advanced Glycation End products
ASCVD	American Heart Association Cardiovascular Disease
AT	Adipose Tissue
BIA	Bioelectrical Impedance Analysis
BMI	Body Mass Index
CAD	Coronary Artery Disease
CABG	Coronary Artery Bypass Graft
CCS	Chronic Coronary Syndrome
CI	Confidence Interval
CKD	Chronic Kidney Disease
CT	Computed Tomography
CV	Cardiovascular
CVD	Cardiovascular Disease
DEXA	Dual-energy X-ray Absorptiometry
DM	Diabetes Mellitus
EMA	European Medicines Agency
ESC	European Society of Cardiology
FDA	Food and Drug Administration
FFAs	Free Fatty Acids
FPG	Fasting Plasma Glucose
GDMT	Guideline Directed Medical Therapy
GIP	Glucose-Dependent Insulinotropic Peptide
GLP-1RAs	Glucagon Like Protein 1 Receptor Agonist
HbA1c	Glycated Hemoglobin
HDL	High-Density Lipoprotein
HF	Heart Failure
HFpEF	Heart Failure with Preserved Ejection Fraction
HFrEF	Heart Failure with Reduced Ejection Fraction
HR	Hazard Ratio
HsCRP	High sensitive C-Reactive Protein
IFN	Interferon
IL	Interleukin
IS	Infarct Size
ISR	In Stent Restenosis
LDL-C	Low-Density Lipoprotein
LLT	Lipid Lowering Therapy
LVEF	Left Ventricular Ejection Fraction
MACE	Major Adverse Cardiovascular Events
MI	Myocardial Infarction
MRI	Magnetic Resonance Imaging
NO	Nitric Oxide
NYHA	New York Heart Association
OMT	Optimal medical therapy
OR	Odds Ratio
PCI	Percutaneous Coronary Intervention
PCSK9-i	Proprotein Convertase Subtilisin/Kexin type 9 Inhibitor
PVAT	PeriVascular Adipose Tissue
RCTs	Randomized Controlled Trials
ROS	Reactive Oxygen Species
RR	Relative Risk
SAT	Subcutaneous Adipose Tissue
SBP	Systolic Blood Pressure
SIL2r	Soluble IL-2 receptor
SMCs	Smooth Muscle Cells
STEMI	ST-up Elevation Myocardial Infarction
T2DM	Type 2 Diabetes Mellitus
TG	Triglycerides
ThAT	Thoracic Adipose Tissue
TNF	Tumor Necrosis Factor
TyG	Triglyceride-Glucose
VAT	Visceral Adipose Tissue
WHO	World Health Organization
